# Charged-particle distributions in *pp* interactions at $\sqrt{s}=8 \mathrm {\, TeV}$ measured with the ATLAS detector

**DOI:** 10.1140/epjc/s10052-016-4203-9

**Published:** 2016-07-15

**Authors:** G. Aad, B. Abbott, J. Abdallah, O. Abdinov, B. Abeloos, R. Aben, M. Abolins, O. S. AbouZeid, N. L. Abraham, H. Abramowicz, H. Abreu, R. Abreu, Y. Abulaiti, B. S. Acharya, L. Adamczyk, D. L. Adams, J. Adelman, S. Adomeit, T. Adye, A. A. Affolder, T. Agatonovic-Jovin, J. Agricola, J. A. Aguilar-Saavedra, S. P. Ahlen, F. Ahmadov, G. Aielli, H. Akerstedt, T. P. A. Åkesson, A. V. Akimov, G. L. Alberghi, J. Albert, S. Albrand, M. J. Alconada Verzini, M. Aleksa, I. N. Aleksandrov, C. Alexa, G. Alexander, T. Alexopoulos, M. Alhroob, M. Aliev, G. Alimonti, J. Alison, S. P. Alkire, B. M. M. Allbrooke, B. W. Allen, P. P. Allport, A. Aloisio, A. Alonso, F. Alonso, C. Alpigiani, B. Alvarez Gonzalez, D. Álvarez Piqueras, M. G. Alviggi, B. T. Amadio, K. Amako, Y. Amaral Coutinho, C. Amelung, D. Amidei, S. P. Amor Dos Santos, A. Amorim, S. Amoroso, N. Amram, G. Amundsen, C. Anastopoulos, L. S. Ancu, N. Andari, T. Andeen, C. F. Anders, G. Anders, J. K. Anders, K. J. Anderson, A. Andreazza, V. Andrei, S. Angelidakis, I. Angelozzi, P. Anger, A. Angerami, F. Anghinolfi, A. V. Anisenkov, N. Anjos, A. Annovi, M. Antonelli, A. Antonov, J. Antos, F. Anulli, M. Aoki, L. Aperio Bella, G. Arabidze, Y. Arai, J. P. Araque, A. T. H. Arce, F. A. Arduh, J-F. Arguin, S. Argyropoulos, M. Arik, A. J. Armbruster, L. J. Armitage, O. Arnaez, H. Arnold, M. Arratia, O. Arslan, A. Artamonov, G. Artoni, S. Artz, S. Asai, N. Asbah, A. Ashkenazi, B. Åsman, L. Asquith, K. Assamagan, R. Astalos, M. Atkinson, N. B. Atlay, K. Augsten, G. Avolio, B. Axen, M. K. Ayoub, G. Azuelos, M. A. Baak, A. E. Baas, M. J. Baca, H. Bachacou, K. Bachas, M. Backes, M. Backhaus, P. Bagiacchi, P. Bagnaia, Y. Bai, J. T. Baines, O. K. Baker, E. M. Baldin, P. Balek, T. Balestri, F. Balli, W. K. Balunas, E. Banas, Sw. Banerjee, A. A. E. Bannoura, L. Barak, E. L. Barberio, D. Barberis, M. Barbero, T. Barillari, M. Barisonzi, T. Barklow, N. Barlow, S. L. Barnes, B. M. Barnett, R. M. Barnett, Z. Barnovska, A. Baroncelli, G. Barone, A. J. Barr, L. Barranco Navarro, F. Barreiro, J. Barreiro Guimarães da Costa, R. Bartoldus, A. E. Barton, P. Bartos, A. Basalaev, A. Bassalat, A. Basye, R. L. Bates, S. J. Batista, J. R. Batley, M. Battaglia, M. Bauce, F. Bauer, H. S. Bawa, J. B. Beacham, M. D. Beattie, T. Beau, P. H. Beauchemin, P. Bechtle, H. P. Beck, K. Becker, M. Becker, M. Beckingham, C. Becot, A. J. Beddall, A. Beddall, V. A. Bednyakov, M. Bedognetti, C. P. Bee, L. J. Beemster, T. A. Beermann, M. Begel, J. K. Behr, C. Belanger-Champagne, A. S. Bell, W. H. Bell, G. Bella, L. Bellagamba, A. Bellerive, M. Bellomo, K. Belotskiy, O. Beltramello, N. L. Belyaev, O. Benary, D. Benchekroun, M. Bender, K. Bendtz, N. Benekos, Y. Benhammou, E. Benhar Noccioli, J. Benitez, J. A. Benitez Garcia, D. P. Benjamin, J. R. Bensinger, S. Bentvelsen, L. Beresford, M. Beretta, D. Berge, E. Bergeaas Kuutmann, N. Berger, F. Berghaus, J. Beringer, S. Berlendis, N. R. Bernard, C. Bernius, F. U. Bernlochner, T. Berry, P. Berta, C. Bertella, G. Bertoli, F. Bertolucci, I. A. Bertram, C. Bertsche, D. Bertsche, G. J. Besjes, O. Bessidskaia Bylund, M. Bessner, N. Besson, C. Betancourt, S. Bethke, A. J. Bevan, W. Bhimji, R. M. Bianchi, L. Bianchini, M. Bianco, O. Biebel, D. Biedermann, R. Bielski, N. V. Biesuz, M. Biglietti, J. Bilbao De Mendizabal, H. Bilokon, M. Bindi, S. Binet, A. Bingul, C. Bini, S. Biondi, D. M. Bjergaard, C. W. Black, J. E. Black, K. M. Black, D. Blackburn, R. E. Blair, J.-B. Blanchard, J. E. Blanco, T. Blazek, I. Bloch, C. Blocker, W. Blum, U. Blumenschein, S. Blunier, G. J. Bobbink, V. S. Bobrovnikov, S. S. Bocchetta, A. Bocci, C. Bock, M. Boehler, D. Boerner, J. A. Bogaerts, D. Bogavac, A. G. Bogdanchikov, C. Bohm, V. Boisvert, T. Bold, V. Boldea, A. S. Boldyrev, M. Bomben, M. Bona, M. Boonekamp, A. Borisov, G. Borissov, J. Bortfeldt, D. Bortoletto, V. Bortolotto, K. Bos, D. Boscherini, M. Bosman, J. D. Bossio Sola, J. Boudreau, J. Bouffard, E. V. Bouhova-Thacker, D. Boumediene, C. Bourdarios, S. K. Boutle, A. Boveia, J. Boyd, I. R. Boyko, J. Bracinik, A. Brandt, G. Brandt, O. Brandt, U. Bratzler, B. Brau, J. E. Brau, H. M. Braun, W. D. Breaden Madden, K. Brendlinger, A. J. Brennan, L. Brenner, R. Brenner, S. Bressler, T. M. Bristow, D. Britton, D. Britzger, F. M. Brochu, I. Brock, R. Brock, G. Brooijmans, T. Brooks, W. K. Brooks, J. Brosamer, E. Brost, J. H Broughton, P. A. Bruckman de Renstrom, D. Bruncko, R. Bruneliere, A. Bruni, G. Bruni, BH Brunt, M. Bruschi, N. Bruscino, P. Bryant, L. Bryngemark, T. Buanes, Q. Buat, P. Buchholz, A. G. Buckley, I. A. Budagov, F. Buehrer, M. K. Bugge, O. Bulekov, D. Bullock, H. Burckhart, S. Burdin, C. D. Burgard, B. Burghgrave, K. Burka, S. Burke, I. Burmeister, E. Busato, D. Büscher, V. Büscher, P. Bussey, J. M. Butler, A. I. Butt, C. M. Buttar, J. M. Butterworth, P. Butti, W. Buttinger, A. Buzatu, A. R. Buzykaev, S. Cabrera Urbán, D. Caforio, V. M. Cairo, O. Cakir, N. Calace, P. Calafiura, A. Calandri, G. Calderini, P. Calfayan, L. P. Caloba, D. Calvet, S. Calvet, T. P. Calvet, R. Camacho Toro, S. Camarda, P. Camarri, D. Cameron, R. Caminal Armadans, C. Camincher, S. Campana, M. Campanelli, A. Campoverde, V. Canale, A. Canepa, M. Cano Bret, J. Cantero, R. Cantrill, T. Cao, M. D. M. Capeans Garrido, I. Caprini, M. Caprini, M. Capua, R. Caputo, R. M. Carbone, R. Cardarelli, F. Cardillo, T. Carli, G. Carlino, L. Carminati, S. Caron, E. Carquin, G. D. Carrillo-Montoya, J. R. Carter, J. Carvalho, D. Casadei, M. P. Casado, M. Casolino, D. W. Casper, E. Castaneda-Miranda, A. Castelli, V. Castillo Gimenez, N. F. Castro, A. Catinaccio, J. R. Catmore, A. Cattai, J. Caudron, V. Cavaliere, E. Cavallaro, D. Cavalli, M. Cavalli-Sforza, V. Cavasinni, F. Ceradini, L. Cerda Alberich, B. C. Cerio, A. S. Cerqueira, A. Cerri, L. Cerrito, F. Cerutti, M. Cerv, A. Cervelli, S. A. Cetin, A. Chafaq, D. Chakraborty, I. Chalupkova, S. K. Chan, Y. L. Chan, P. Chang, J. D. Chapman, D. G. Charlton, A. Chatterjee, C. C. Chau, C. A. Chavez Barajas, S. Che, S. Cheatham, A. Chegwidden, S. Chekanov, S. V. Chekulaev, G. A. Chelkov, M. A. Chelstowska, C. Chen, H. Chen, K. Chen, S. Chen, S. Chen, X. Chen, Y. Chen, H. C. Cheng, H. J Cheng, Y. Cheng, A. Cheplakov, E. Cheremushkina, R. Cherkaoui El Moursli, V. Chernyatin, E. Cheu, L. Chevalier, V. Chiarella, G. Chiarelli, G. Chiodini, A. S. Chisholm, A. Chitan, M. V. Chizhov, K. Choi, A. R. Chomont, S. Chouridou, B. K. B. Chow, V. Christodoulou, D. Chromek-Burckhart, J. Chudoba, A. J. Chuinard, J. J. Chwastowski, L. Chytka, G. Ciapetti, A. K. Ciftci, D. Cinca, V. Cindro, I. A. Cioara, A. Ciocio, F. Cirotto, Z. H. Citron, M. Ciubancan, A. Clark, B. L. Clark, M. R. Clark, P. J. Clark, R. N. Clarke, C. Clement, Y. Coadou, M. Cobal, A. Coccaro, J. Cochran, L. Coffey, L. Colasurdo, B. Cole, S. Cole, A. P. Colijn, J. Collot, T. Colombo, G. Compostella, P. Conde Muiño, E. Coniavitis, S. H. Connell, I. A. Connelly, V. Consorti, S. Constantinescu, C. Conta, G. Conti, F. Conventi, M. Cooke, B. D. Cooper, A. M. Cooper-Sarkar, T. Cornelissen, M. Corradi, F. Corriveau, A. Corso-Radu, A. Cortes-Gonzalez, G. Cortiana, G. Costa, M. J. Costa, D. Costanzo, G. Cottin, G. Cowan, B. E. Cox, K. Cranmer, S. J. Crawley, G. Cree, S. Crépé-Renaudin, F. Crescioli, W. A. Cribbs, M. Crispin Ortuzar, M. Cristinziani, V. Croft, G. Crosetti, T. Cuhadar Donszelmann, J. Cummings, M. Curatolo, J. Cúth, C. Cuthbert, H. Czirr, P. Czodrowski, S. D’Auria, M. D’Onofrio, M. J. Da Cunha Sargedas De Sousa, C. Da Via, W. Dabrowski, T. Dai, O. Dale, F. Dallaire, C. Dallapiccola, M. Dam, J. R. Dandoy, N. P. Dang, A. C. Daniells, N. S. Dann, M. Danninger, M. Dano Hoffmann, V. Dao, G. Darbo, S. Darmora, J. Dassoulas, A. Dattagupta, W. Davey, C. David, T. Davidek, M. Davies, P. Davison, Y. Davygora, E. Dawe, I. Dawson, R. K. Daya-Ishmukhametova, K. De, R. de Asmundis, A. De Benedetti, S. De Castro, S. De Cecco, N. De Groot, P. de Jong, H. De la Torre, F. De Lorenzi, D. De Pedis, A. De Salvo, U. De Sanctis, A. De Santo, J. B. De Vivie De Regie, W. J. Dearnaley, R. Debbe, C. Debenedetti, D. V. Dedovich, I. Deigaard, J. Del Peso, T. Del Prete, D. Delgove, F. Deliot, C. M. Delitzsch, M. Deliyergiyev, A. Dell’Acqua, L. Dell’Asta, M. Dell’Orso, M. Della Pietra, D. della Volpe, M. Delmastro, P. A. Delsart, C. Deluca, D. A. DeMarco, S. Demers, M. Demichev, A. Demilly, S. P. Denisov, D. Denysiuk, D. Derendarz, J. E. Derkaoui, F. Derue, P. Dervan, K. Desch, C. Deterre, K. Dette, P. O. Deviveiros, A. Dewhurst, S. Dhaliwal, A. Di Ciaccio, L. Di Ciaccio, W. K. Di Clemente, A. Di Domenico, C. Di Donato, A. Di Girolamo, B. Di Girolamo, A. Di Mattia, B. Di Micco, R. Di Nardo, A. Di Simone, R. Di Sipio, D. Di Valentino, C. Diaconu, M. Diamond, F. A. Dias, M. A. Diaz, E. B. Diehl, J. Dietrich, S. Diglio, A. Dimitrievska, J. Dingfelder, P. Dita, S. Dita, F. Dittus, F. Djama, T. Djobava, J. I. Djuvsland, M. A. B. do Vale, D. Dobos, M. Dobre, C. Doglioni, T. Dohmae, J. Dolejsi, Z. Dolezal, B. A. Dolgoshein, M. Donadelli, S. Donati, P. Dondero, J. Donini, J. Dopke, A. Doria, M. T. Dova, A. T. Doyle, E. Drechsler, M. Dris, Y. Du, J. Duarte-Campderros, E. Duchovni, G. Duckeck, O. A. Ducu, D. Duda, A. Dudarev, L. Duflot, L. Duguid, M. Dührssen, M. Dunford, H. Duran Yildiz, M. Düren, A. Durglishvili, D. Duschinger, B. Dutta, M. Dyndal, C. Eckardt, K. M. Ecker, R. C. Edgar, W. Edson, N. C. Edwards, T. Eifert, G. Eigen, K. Einsweiler, T. Ekelof, M. El Kacimi, V. Ellajosyula, M. Ellert, S. Elles, F. Ellinghaus, A. A. Elliot, N. Ellis, J. Elmsheuser, M. Elsing, D. Emeliyanov, Y. Enari, O. C. Endner, M. Endo, J. S. Ennis, J. Erdmann, A. Ereditato, G. Ernis, J. Ernst, M. Ernst, S. Errede, E. Ertel, M. Escalier, H. Esch, C. Escobar, B. Esposito, A. I. Etienvre, E. Etzion, H. Evans, A. Ezhilov, F. Fabbri, L. Fabbri, G. Facini, R. M. Fakhrutdinov, S. Falciano, R. J. Falla, J. Faltova, Y. Fang, M. Fanti, A. Farbin, A. Farilla, C. Farina, T. Farooque, S. Farrell, S. M. Farrington, P. Farthouat, F. Fassi, P. Fassnacht, D. Fassouliotis, M. Faucci Giannelli, A. Favareto, W. J. Fawcett, L. Fayard, O. L. Fedin, W. Fedorko, S. Feigl, L. Feligioni, C. Feng, E. J. Feng, H. Feng, A. B. Fenyuk, L. Feremenga, P. Fernandez Martinez, S. Fernandez Perez, J. Ferrando, A. Ferrari, P. Ferrari, R. Ferrari, D. E. Ferreira de Lima, A. Ferrer, D. Ferrere, C. Ferretti, A. Ferretto Parodi, F. Fiedler, A. Filipčič, M. Filipuzzi, F. Filthaut, M. Fincke-Keeler, K. D. Finelli, M. C. N. Fiolhais, L. Fiorini, A. Firan, A. Fischer, C. Fischer, J. Fischer, W. C. Fisher, N. Flaschel, I. Fleck, P. Fleischmann, G. T. Fletcher, G. Fletcher, R. R. M. Fletcher, T. Flick, A. Floderus, L. R. Flores Castillo, M. J. Flowerdew, G. T. Forcolin, A. Formica, A. Forti, A. G. Foster, D. Fournier, H. Fox, S. Fracchia, P. Francavilla, M. Franchini, D. Francis, L. Franconi, M. Franklin, M. Frate, M. Fraternali, D. Freeborn, S. M. Fressard-Batraneanu, F. Friedrich, D. Froidevaux, J. A. Frost, C. Fukunaga, E. Fullana Torregrosa, T. Fusayasu, J. Fuster, C. Gabaldon, O. Gabizon, A. Gabrielli, A. Gabrielli, G. P. Gach, S. Gadatsch, S. Gadomski, G. Gagliardi, L. G. Gagnon, P. Gagnon, C. Galea, B. Galhardo, E. J. Gallas, B. J. Gallop, P. Gallus, G. Galster, K. K. Gan, J. Gao, Y. Gao, Y. S. Gao, F. M. Garay Walls, C. García, J. E. García Navarro, M. Garcia-Sciveres, R. W. Gardner, N. Garelli, V. Garonne, A. Gascon Bravo, C. Gatti, A. Gaudiello, G. Gaudio, B. Gaur, L. Gauthier, I. L. Gavrilenko, C. Gay, G. Gaycken, E. N. Gazis, Z. Gecse, C. N. P. Gee, Ch. Geich-Gimbel, M. P. Geisler, C. Gemme, M. H. Genest, C. Geng, S. Gentile, S. George, D. Gerbaudo, A. Gershon, S. Ghasemi, H. Ghazlane, M. Ghneimat, B. Giacobbe, S. Giagu, P. Giannetti, B. Gibbard, S. M. Gibson, M. Gignac, M. Gilchriese, T. P. S. Gillam, D. Gillberg, G. Gilles, D. M. Gingrich, N. Giokaris, M. P. Giordani, F. M. Giorgi, F. M. Giorgi, P. F. Giraud, P. Giromini, D. Giugni, F. Giuli, C. Giuliani, M. Giulini, B. K. Gjelsten, S. Gkaitatzis, I. Gkialas, E. L. Gkougkousis, L. K. Gladilin, C. Glasman, J. Glatzer, P. C. F. Glaysher, A. Glazov, M. Goblirsch-Kolb, J. Godlewski, S. Goldfarb, T. Golling, D. Golubkov, A. Gomes, R. Gonçalo, J. Goncalves Pinto Firmino Da Costa, L. Gonella, A. Gongadze, S. González de la Hoz, G. Gonzalez Parra, S. Gonzalez-Sevilla, L. Goossens, P. A. Gorbounov, H. A. Gordon, I. Gorelov, B. Gorini, E. Gorini, A. Gorišek, E. Gornicki, A. T. Goshaw, C. Gössling, M. I. Gostkin, C. R. Goudet, D. Goujdami, A. G. Goussiou, N. Govender, E. Gozani, L. Graber, I. Grabowska-Bold, P. O. J. Gradin, P. Grafström, J. Gramling, E. Gramstad, S. Grancagnolo, V. Gratchev, H. M. Gray, E. Graziani, Z. D. Greenwood, C. Grefe, K. Gregersen, I. M. Gregor, P. Grenier, K. Grevtsov, J. Griffiths, A. A. Grillo, K. Grimm, S. Grinstein, Ph. Gris, J.-F. Grivaz, S. Groh, J. P. Grohs, E. Gross, J. Grosse-Knetter, G. C. Grossi, Z. J. Grout, L. Guan, W. Guan, J. Guenther, F. Guescini, D. Guest, O. Gueta, E. Guido, T. Guillemin, S. Guindon, U. Gul, C. Gumpert, J. Guo, Y. Guo, S. Gupta, G. Gustavino, P. Gutierrez, N. G. Gutierrez Ortiz, C. Gutschow, C. Guyot, C. Gwenlan, C. B. Gwilliam, A. Haas, C. Haber, H. K. Hadavand, N. Haddad, A. Hadef, P. Haefner, S. Hageböck, Z. Hajduk, H. Hakobyan, M. Haleem, J. Haley, D. Hall, G. Halladjian, G. D. Hallewell, K. Hamacher, P. Hamal, K. Hamano, A. Hamilton, G. N. Hamity, P. G. Hamnett, L. Han, K. Hanagaki, K. Hanawa, M. Hance, B. Haney, P. Hanke, R. Hanna, J. B. Hansen, J. D. Hansen, M. C. Hansen, P. H. Hansen, K. Hara, A. S. Hard, T. Harenberg, F. Hariri, S. Harkusha, R. D. Harrington, P. F. Harrison, F. Hartjes, M. Hasegawa, Y. Hasegawa, A. Hasib, S. Hassani, S. Haug, R. Hauser, L. Hauswald, M. Havranek, C. M. Hawkes, R. J. Hawkings, A. D. Hawkins, D. Hayden, C. P. Hays, J. M. Hays, H. S. Hayward, S. J. Haywood, S. J. Head, T. Heck, V. Hedberg, L. Heelan, S. Heim, T. Heim, B. Heinemann, J. J. Heinrich, L. Heinrich, C. Heinz, J. Hejbal, L. Helary, S. Hellman, C. Helsens, J. Henderson, R. C. W. Henderson, Y. Heng, S. Henkelmann, A. M. Henriques Correia, S. Henrot-Versille, G. H. Herbert, Y. Hernández Jiménez, G. Herten, R. Hertenberger, L. Hervas, G. G. Hesketh, N. P. Hessey, J. W. Hetherly, R. Hickling, E. Higón-Rodriguez, E. Hill, J. C. Hill, K. H. Hiller, S. J. Hillier, I. Hinchliffe, E. Hines, R. R. Hinman, M. Hirose, D. Hirschbuehl, J. Hobbs, N. Hod, M. C. Hodgkinson, P. Hodgson, A. Hoecker, M. R. Hoeferkamp, F. Hoenig, M. Hohlfeld, D. Hohn, T. R. Holmes, M. Homann, T. M. Hong, B. H. Hooberman, W. H. Hopkins, Y. Horii, A. J. Horton, J-Y. Hostachy, S. Hou, A. Hoummada, J. Howard, J. Howarth, M. Hrabovsky, I. Hristova, J. Hrivnac, T. Hryn’ova, A. Hrynevich, C. Hsu, P. J. Hsu, S.-C. Hsu, D. Hu, Q. Hu, Y. Huang, Z. Hubacek, F. Hubaut, F. Huegging, T. B. Huffman, E. W. Hughes, G. Hughes, M. Huhtinen, T. A. Hülsing, N. Huseynov, J. Huston, J. Huth, G. Iacobucci, G. Iakovidis, I. Ibragimov, L. Iconomidou-Fayard, E. Ideal, Z. Idrissi, P. Iengo, O. Igonkina, T. Iizawa, Y. Ikegami, M. Ikeno, Y. Ilchenko, D. Iliadis, N. Ilic, T. Ince, G. Introzzi, P. Ioannou, M. Iodice, K. Iordanidou, V. Ippolito, A. Irles Quiles, C. Isaksson, M. Ishino, M. Ishitsuka, R. Ishmukhametov, C. Issever, S. Istin, F. Ito, J. M. Iturbe Ponce, R. Iuppa, J. Ivarsson, W. Iwanski, H. Iwasaki, J. M. Izen, V. Izzo, S. Jabbar, B. Jackson, M. Jackson, P. Jackson, V. Jain, K. B. Jakobi, K. Jakobs, S. Jakobsen, T. Jakoubek, D. O. Jamin, D. K. Jana, E. Jansen, R. Jansky, J. Janssen, M. Janus, G. Jarlskog, N. Javadov, T. Javůrek, F. Jeanneau, L. Jeanty, J. Jejelava, G.-Y. Jeng, D. Jennens, P. Jenni, J. Jentzsch, C. Jeske, S. Jézéquel, H. Ji, J. Jia, H. Jiang, Y. Jiang, S. Jiggins, J. Jimenez Pena, S. Jin, A. Jinaru, O. Jinnouchi, P. Johansson, K. A. Johns, W. J. Johnson, K. Jon-And, G. Jones, R. W. L. Jones, S. Jones, T. J. Jones, J. Jongmanns, P. M. Jorge, J. Jovicevic, X. Ju, A. Juste Rozas, M. K. Köhler, A. Kaczmarska, M. Kado, H. Kagan, M. Kagan, S. J. Kahn, E. Kajomovitz, C. W. Kalderon, A. Kaluza, S. Kama, A. Kamenshchikov, N. Kanaya, S. Kaneti, L. Kanjir, V. A. Kantserov, J. Kanzaki, B. Kaplan, L. S. Kaplan, A. Kapliy, D. Kar, K. Karakostas, A. Karamaoun, N. Karastathis, M. J. Kareem, E. Karentzos, M. Karnevskiy, S. N. Karpov, Z. M. Karpova, K. Karthik, V. Kartvelishvili, A. N. Karyukhin, K. Kasahara, L. Kashif, R. D. Kass, A. Kastanas, Y. Kataoka, C. Kato, A. Katre, J. Katzy, K. Kawade, K. Kawagoe, T. Kawamoto, G. Kawamura, S. Kazama, V. F. Kazanin, R. Keeler, R. Kehoe, J. S. Keller, J. J. Kempster, H. Keoshkerian, O. Kepka, B. P. Kerševan, S. Kersten, R. A. Keyes, F. Khalil-zada, H. Khandanyan, A. Khanov, A. G. Kharlamov, T. J. Khoo, V. Khovanskiy, E. Khramov, J. Khubua, S. Kido, H. Y. Kim, S. H. Kim, Y. K. Kim, N. Kimura, O. M. Kind, B. T. King, M. King, S. B. King, J. Kirk, A. E. Kiryunin, T. Kishimoto, D. Kisielewska, F. Kiss, K. Kiuchi, O. Kivernyk, E. Kladiva, M. H. Klein, M. Klein, U. Klein, K. Kleinknecht, P. Klimek, A. Klimentov, R. Klingenberg, J. A. Klinger, T. Klioutchnikova, E.-E. Kluge, P. Kluit, S. Kluth, J. Knapik, E. Kneringer, E. B. F. G. Knoops, A. Knue, A. Kobayashi, D. Kobayashi, T. Kobayashi, M. Kobel, M. Kocian, P. Kodys, T. Koffas, E. Koffeman, L. A. Kogan, T. Kohriki, T. Koi, H. Kolanoski, M. Kolb, I. Koletsou, A. A. Komar, Y. Komori, T. Kondo, N. Kondrashova, K. Köneke, A. C. König, T. Kono, R. Konoplich, N. Konstantinidis, R. Kopeliansky, S. Koperny, L. Köpke, A. K. Kopp, K. Korcyl, K. Kordas, A. Korn, A. A. Korol, I. Korolkov, E. V. Korolkova, O. Kortner, S. Kortner, T. Kosek, V. V. Kostyukhin, V. M. Kotov, A. Kotwal, A. Kourkoumeli-Charalampidi, C. Kourkoumelis, V. Kouskoura, A. Koutsman, A. B. Kowalewska, R. Kowalewski, T. Z. Kowalski, W. Kozanecki, A. S. Kozhin, V. A. Kramarenko, G. Kramberger, D. Krasnopevtsev, M. W. Krasny, A. Krasznahorkay, J. K. Kraus, A. Kravchenko, M. Kretz, J. Kretzschmar, K. Kreutzfeldt, P. Krieger, K. Krizka, K. Kroeninger, H. Kroha, J. Kroll, J. Kroseberg, J. Krstic, U. Kruchonak, H. Krüger, N. Krumnack, A. Kruse, M. C. Kruse, M. Kruskal, T. Kubota, H. Kucuk, S. Kuday, J. T. Kuechler, S. Kuehn, A. Kugel, F. Kuger, A. Kuhl, T. Kuhl, V. Kukhtin, R. Kukla, Y. Kulchitsky, S. Kuleshov, M. Kuna, T. Kunigo, A. Kupco, H. Kurashige, Y. A. Kurochkin, V. Kus, E. S. Kuwertz, M. Kuze, J. Kvita, T. Kwan, D. Kyriazopoulos, A. La Rosa, J. L. La Rosa Navarro, L. La Rotonda, C. Lacasta, F. Lacava, J. Lacey, H. Lacker, D. Lacour, V. R. Lacuesta, E. Ladygin, R. Lafaye, B. Laforge, T. Lagouri, S. Lai, S. Lammers, W. Lampl, E. Lançon, U. Landgraf, M. P. J. Landon, V. S. Lang, J. C. Lange, A. J. Lankford, F. Lanni, K. Lantzsch, A. Lanza, S. Laplace, C. Lapoire, J. F. Laporte, T. Lari, F. Lasagni Manghi, M. Lassnig, P. Laurelli, W. Lavrijsen, A. T. Law, P. Laycock, T. Lazovich, M. Lazzaroni, O. Le Dortz, E. Le Guirriec, E. Le Menedeu, E. P. Le Quilleuc, M. LeBlanc, T. LeCompte, F. Ledroit-Guillon, C. A. Lee, S. C. Lee, L. Lee, G. Lefebvre, M. Lefebvre, F. Legger, C. Leggett, A. Lehan, G. Lehmann Miotto, X. Lei, W. A. Leight, A. Leisos, A. G. Leister, M. A. L. Leite, R. Leitner, D. Lellouch, B. Lemmer, K. J. C. Leney, T. Lenz, B. Lenzi, R. Leone, S. Leone, C. Leonidopoulos, S. Leontsinis, G. Lerner, C. Leroy, A. A. J. Lesage, C. G. Lester, M. Levchenko, J. Levêque, D. Levin, L. J. Levinson, M. Levy, A. M. Leyko, M. Leyton, B. Li, H. Li, H. L. Li, L. Li, L. Li, Q. Li, S. Li, X. Li, Y. Li, Z. Liang, H. Liao, B. Liberti, A. Liblong, P. Lichard, K. Lie, J. Liebal, W. Liebig, C. Limbach, A. Limosani, S. C. Lin, T. H. Lin, B. E. Lindquist, E. Lipeles, A. Lipniacka, M. Lisovyi, T. M. Liss, D. Lissauer, A. Lister, A. M. Litke, B. Liu, D. Liu, H. Liu, H. Liu, J. Liu, J. B. Liu, K. Liu, L. Liu, M. Liu, M. Liu, Y. L. Liu, Y. Liu, M. Livan, A. Lleres, J. Llorente Merino, S. L. Lloyd, F. Lo Sterzo, E. Lobodzinska, P. Loch, W. S. Lockman, F. K. Loebinger, A. E. Loevschall-Jensen, K. M. Loew, A. Loginov, T. Lohse, K. Lohwasser, M. Lokajicek, B. A. Long, J. D. Long, R. E. Long, L. Longo, K. A. Looper, L. Lopes, D. Lopez Mateos, B. Lopez Paredes, I. Lopez Paz, A. Lopez Solis, J. Lorenz, N. Lorenzo Martinez, M. Losada, P. J. Lösel, X. Lou, A. Lounis, J. Love, P. A. Love, H. Lu, N. Lu, H. J. Lubatti, C. Luci, A. Lucotte, C. Luedtke, F. Luehring, W. Lukas, L. Luminari, O. Lundberg, B. Lund-Jensen, D. Lynn, R. Lysak, E. Lytken, V. Lyubushkin, H. Ma, L. L. Ma, Y. Ma, G. Maccarrone, A. Macchiolo, C. M. Macdonald, B. Maček, J. Machado Miguens, D. Madaffari, R. Madar, H. J. Maddocks, W. F. Mader, A. Madsen, J. Maeda, S. Maeland, T. Maeno, A. Maevskiy, E. Magradze, J. Mahlstedt, C. Maiani, C. Maidantchik, A. A. Maier, T. Maier, A. Maio, S. Majewski, Y. Makida, N. Makovec, B. Malaescu, Pa. Malecki, V. P. Maleev, F. Malek, U. Mallik, D. Malon, C. Malone, S. Maltezos, V. M. Malyshev, S. Malyukov, J. Mamuzic, G. Mancini, B. Mandelli, L. Mandelli, I. Mandić, J. Maneira, L. Manhaes de Andrade Filho, J. Manjarres Ramos, A. Mann, B. Mansoulie, R. Mantifel, M. Mantoani, S. Manzoni, L. Mapelli, G. Marceca, L. March, G. Marchiori, M. Marcisovsky, M. Marjanovic, D. E. Marley, F. Marroquim, S. P. Marsden, Z. Marshall, L. F. Marti, S. Marti-Garcia, B. Martin, T. A. Martin, V. J. Martin, B. Martin dit Latour, M. Martinez, S. Martin-Haugh, V. S. Martoiu, A. C. Martyniuk, M. Marx, F. Marzano, A. Marzin, L. Masetti, T. Mashimo, R. Mashinistov, J. Masik, A. L. Maslennikov, I. Massa, L. Massa, P. Mastrandrea, A. Mastroberardino, T. Masubuchi, P. Mättig, J. Mattmann, J. Maurer, S. J. Maxfield, D. A. Maximov, R. Mazini, S. M. Mazza, N. C. Mc Fadden, G. Mc Goldrick, S. P. Mc Kee, A. McCarn, R. L. McCarthy, T. G. McCarthy, L. I. McClymont, K. W. McFarlane, J. A. Mcfayden, G. Mchedlidze, S. J. McMahon, R. A. McPherson, M. Medici, M. Medinnis, S. Meehan, S. Mehlhase, A. Mehta, K. Meier, C. Meineck, B. Meirose, B. R. Mellado Garcia, F. Meloni, A. Mengarelli, S. Menke, E. Meoni, K. M. Mercurio, S. Mergelmeyer, P. Mermod, L. Merola, C. Meroni, F. S. Merritt, A. Messina, J. Metcalfe, A. S. Mete, C. Meyer, C. Meyer, J-P. Meyer, J. Meyer, H. Meyer Zu Theenhausen, R. P. Middleton, S. Miglioranzi, L. Mijović, G. Mikenberg, M. Mikestikova, M. Mikuž, M. Milesi, A. Milic, D. W. Miller, C. Mills, A. Milov, D. A. Milstead, A. A. Minaenko, Y. Minami, I. A. Minashvili, A. I. Mincer, B. Mindur, M. Mineev, Y. Ming, L. M. Mir, K. P. Mistry, T. Mitani, J. Mitrevski, V. A. Mitsou, A. Miucci, P. S. Miyagawa, J. U. Mjörnmark, T. Moa, K. Mochizuki, S. Mohapatra, W. Mohr, S. Molander, R. Moles-Valls, R. Monden, M. C. Mondragon, K. Mönig, J. Monk, E. Monnier, A. Montalbano, J. Montejo Berlingen, F. Monticelli, S. Monzani, R. W. Moore, N. Morange, D. Moreno, M. Moreno Llácer, P. Morettini, D. Mori, T. Mori, M. Morii, M. Morinaga, V. Morisbak, S. Moritz, A. K. Morley, G. Mornacchi, J. D. Morris, S. S. Mortensen, L. Morvaj, M. Mosidze, J. Moss, K. Motohashi, R. Mount, E. Mountricha, S. V. Mouraviev, E. J. W. Moyse, S. Muanza, R. D. Mudd, F. Mueller, J. Mueller, R. S. P. Mueller, T. Mueller, D. Muenstermann, P. Mullen, G. A. Mullier, F. J. Munoz Sanchez, J. A. Murillo Quijada, W. J. Murray, H. Musheghyan, M. Muskinja, A. G. Myagkov, M. Myska, B. P. Nachman, O. Nackenhorst, J. Nadal, K. Nagai, R. Nagai, K. Nagano, Y. Nagasaka, K. Nagata, M. Nagel, E. Nagy, A. M. Nairz, Y. Nakahama, K. Nakamura, T. Nakamura, I. Nakano, H. Namasivayam, R. F. Naranjo Garcia, R. Narayan, D. I. Narrias Villar, I. Naryshkin, T. Naumann, G. Navarro, R. Nayyar, H. A. Neal, P. Yu. Nechaeva, T. J. Neep, P. D. Nef, A. Negri, M. Negrini, S. Nektarijevic, C. Nellist, A. Nelson, S. Nemecek, P. Nemethy, A. A. Nepomuceno, M. Nessi, M. S. Neubauer, M. Neumann, R. M. Neves, P. Nevski, P. R. Newman, D. H. Nguyen, R. B. Nickerson, R. Nicolaidou, B. Nicquevert, J. Nielsen, A. Nikiforov, V. Nikolaenko, I. Nikolic-Audit, K. Nikolopoulos, J. K. Nilsen, P. Nilsson, Y. Ninomiya, A. Nisati, R. Nisius, T. Nobe, L. Nodulman, M. Nomachi, I. Nomidis, T. Nooney, S. Norberg, M. Nordberg, N. Norjoharuddeen, O. Novgorodova, S. Nowak, M. Nozaki, L. Nozka, K. Ntekas, E. Nurse, F. Nuti, F. O’grady, D. C. O’Neil, A. A. O’Rourke, V. O’Shea, F. G. Oakham, H. Oberlack, T. Obermann, J. Ocariz, A. Ochi, I. Ochoa, J. P. Ochoa-Ricoux, S. Oda, S. Odaka, H. Ogren, A. Oh, S. H. Oh, C. C. Ohm, H. Ohman, H. Oide, H. Okawa, Y. Okumura, T. Okuyama, A. Olariu, L. F. Oleiro Seabra, S. A. Olivares Pino, D. Oliveira Damazio, A. Olszewski, J. Olszowska, A. Onofre, K. Onogi, P. U. E. Onyisi, C. J. Oram, M. J. Oreglia, Y. Oren, D. Orestano, N. Orlando, R. S. Orr, B. Osculati, R. Ospanov, G. Otero y Garzon, H. Otono, M. Ouchrif, F. Ould-Saada, A. Ouraou, K. P. Oussoren, Q. Ouyang, M. Owen, R. E. Owen, V. E. Ozcan, N. Ozturk, K. Pachal, A. Pacheco Pages, C. Padilla Aranda, M. Pagáčová, S. Pagan Griso, F. Paige, P. Pais, K. Pajchel, G. Palacino, S. Palestini, M. Palka, D. Pallin, A. Palma, E. St. Panagiotopoulou, C. E. Pandini, J. G. Panduro Vazquez, P. Pani, S. Panitkin, D. Pantea, L. Paolozzi, Th. D. Papadopoulou, K. Papageorgiou, A. Paramonov, D. Paredes Hernandez, A. J. Parker, M. A. Parker, K. A. Parker, F. Parodi, J. A. Parsons, U. Parzefall, V. R. Pascuzzi, E. Pasqualucci, S. Passaggio, F. Pastore, Fr. Pastore, G. Pásztor, S. Pataraia, N. D. Patel, J. R. Pater, T. Pauly, J. Pearce, B. Pearson, L. E. Pedersen, M. Pedersen, S. Pedraza Lopez, R. Pedro, S. V. Peleganchuk, D. Pelikan, O. Penc, C. Peng, H. Peng, J. Penwell, B. S. Peralva, M. M. Perego, D. V. Perepelitsa, E. Perez Codina, L. Perini, H. Pernegger, S. Perrella, R. Peschke, V. D. Peshekhonov, K. Peters, R. F. Y. Peters, B. A. Petersen, T. C. Petersen, E. Petit, A. Petridis, C. Petridou, P. Petroff, E. Petrolo, M. Petrov, F. Petrucci, N. E. Pettersson, A. Peyaud, R. Pezoa, P. W. Phillips, G. Piacquadio, E. Pianori, A. Picazio, E. Piccaro, M. Piccinini, M. A. Pickering, R. Piegaia, J. E. Pilcher, A. D. Pilkington, A. W. J. Pin, J. Pina, M. Pinamonti, J. L. Pinfold, A. Pingel, S. Pires, H. Pirumov, M. Pitt, L. Plazak, M.-A. Pleier, V. Pleskot, E. Plotnikova, P. Plucinski, D. Pluth, R. Poettgen, L. Poggioli, D. Pohl, G. Polesello, A. Poley, A. Policicchio, R. Polifka, A. Polini, C. S. Pollard, V. Polychronakos, K. Pommès, L. Pontecorvo, B. G. Pope, G. A. Popeneciu, D. S. Popovic, A. Poppleton, S. Pospisil, K. Potamianos, I. N. Potrap, C. J. Potter, C. T. Potter, G. Poulard, J. Poveda, V. Pozdnyakov, M. E. Pozo Astigarraga, P. Pralavorio, A. Pranko, S. Prell, D. Price, L. E. Price, M. Primavera, S. Prince, M. Proissl, K. Prokofiev, F. Prokoshin, S. Protopopescu, J. Proudfoot, M. Przybycien, D. Puddu, D. Puldon, M. Purohit, P. Puzo, J. Qian, G. Qin, Y. Qin, A. Quadt, W. B. Quayle, M. Queitsch-Maitland, D. Quilty, S. Raddum, V. Radeka, V. Radescu, S. K. Radhakrishnan, P. Radloff, P. Rados, F. Ragusa, G. Rahal, J. A. Raine, S. Rajagopalan, M. Rammensee, C. Rangel-Smith, M. G. Ratti, F. Rauscher, S. Rave, T. Ravenscroft, M. Raymond, A. L. Read, N. P. Readioff, D. M. Rebuzzi, A. Redelbach, G. Redlinger, R. Reece, K. Reeves, L. Rehnisch, J. Reichert, H. Reisin, C. Rembser, H. Ren, M. Rescigno, S. Resconi, O. L. Rezanova, P. Reznicek, R. Rezvani, R. Richter, S. Richter, E. Richter-Was, O. Ricken, M. Ridel, P. Rieck, C. J. Riegel, J. Rieger, O. Rifki, M. Rijssenbeek, A. Rimoldi, L. Rinaldi, B. Ristić, E. Ritsch, I. Riu, F. Rizatdinova, E. Rizvi, C. Rizzi, S. H. Robertson, A. Robichaud-Veronneau, D. Robinson, J. E. M. Robinson, A. Robson, C. Roda, Y. Rodina, A. Rodriguez Perez, D. Rodriguez Rodriguez, S. Roe, C. S. Rogan, O. Røhne, A. Romaniouk, M. Romano, S. M. Romano Saez, E. Romero Adam, N. Rompotis, M. Ronzani, L. Roos, E. Ros, S. Rosati, K. Rosbach, P. Rose, O. Rosenthal, V. Rossetti, E. Rossi, L. P. Rossi, J. H. N. Rosten, R. Rosten, M. Rotaru, I. Roth, J. Rothberg, D. Rousseau, C. R. Royon, A. Rozanov, Y. Rozen, X. Ruan, F. Rubbo, I. Rubinskiy, V. I. Rud, M. S. Rudolph, F. Rühr, A. Ruiz-Martinez, Z. Rurikova, N. A. Rusakovich, A. Ruschke, H. L. Russell, J. P. Rutherfoord, N. Ruthmann, Y. F. Ryabov, M. Rybar, G. Rybkin, S. Ryu, A. Ryzhov, A. F. Saavedra, G. Sabato, S. Sacerdoti, H. F-W. Sadrozinski, R. Sadykov, F. Safai Tehrani, P. Saha, M. Sahinsoy, M. Saimpert, T. Saito, H. Sakamoto, Y. Sakurai, G. Salamanna, A. Salamon, J. E. Salazar Loyola, D. Salek, P. H. Sales De Bruin, D. Salihagic, A. Salnikov, J. Salt, D. Salvatore, F. Salvatore, A. Salvucci, A. Salzburger, D. Sammel, D. Sampsonidis, A. Sanchez, J. Sánchez, V. Sanchez Martinez, H. Sandaker, R. L. Sandbach, H. G. Sander, M. P. Sanders, M. Sandhoff, C. Sandoval, R. Sandstroem, D. P. C. Sankey, M. Sannino, A. Sansoni, C. Santoni, R. Santonico, H. Santos, I. Santoyo Castillo, K. Sapp, A. Sapronov, J. G. Saraiva, B. Sarrazin, O. Sasaki, Y. Sasaki, K. Sato, G. Sauvage, E. Sauvan, G. Savage, P. Savard, C. Sawyer, L. Sawyer, J. Saxon, C. Sbarra, A. Sbrizzi, T. Scanlon, D. A. Scannicchio, M. Scarcella, V. Scarfone, J. Schaarschmidt, P. Schacht, D. Schaefer, R. Schaefer, J. Schaeffer, S. Schaepe, S. Schaetzel, U. Schäfer, A. C. Schaffer, D. Schaile, R. D. Schamberger, V. Scharf, V. A. Schegelsky, D. Scheirich, M. Schernau, C. Schiavi, C. Schillo, M. Schioppa, S. Schlenker, K. Schmieden, C. Schmitt, S. Schmitt, S. Schmitz, B. Schneider, Y. J. Schnellbach, U. Schnoor, L. Schoeffel, A. Schoening, B. D. Schoenrock, E. Schopf, A. L. S. Schorlemmer, M. Schott, J. Schovancova, S. Schramm, M. Schreyer, N. Schuh, M. J. Schultens, H.-C. Schultz-Coulon, H. Schulz, M. Schumacher, B. A. Schumm, Ph. Schune, C. Schwanenberger, A. Schwartzman, T. A. Schwarz, Ph. Schwegler, H. Schweiger, Ph. Schwemling, R. Schwienhorst, J. Schwindling, T. Schwindt, G. Sciolla, F. Scuri, F. Scutti, J. Searcy, P. Seema, S. C. Seidel, A. Seiden, F. Seifert, J. M. Seixas, G. Sekhniaidze, K. Sekhon, S. J. Sekula, D. M. Seliverstov, N. Semprini-Cesari, C. Serfon, L. Serin, L. Serkin, M. Sessa, R. Seuster, H. Severini, T. Sfiligoj, F. Sforza, A. Sfyrla, E. Shabalina, N. W. Shaikh, L. Y. Shan, R. Shang, J. T. Shank, M. Shapiro, P. B. Shatalov, K. Shaw, S. M. Shaw, A. Shcherbakova, C. Y. Shehu, P. Sherwood, L. Shi, S. Shimizu, C. O. Shimmin, M. Shimojima, M. Shiyakova, A. Shmeleva, D. Shoaleh Saadi, M. J. Shochet, S. Shojaii, S. Shrestha, E. Shulga, M. A. Shupe, P. Sicho, P. E. Sidebo, O. Sidiropoulou, D. Sidorov, A. Sidoti, F. Siegert, Dj. Sijacki, J. Silva, S. B. Silverstein, V. Simak, O. Simard, Lj. Simic, S. Simion, E. Simioni, B. Simmons, D. Simon, M. Simon, P. Sinervo, N. B. Sinev, M. Sioli, G. Siragusa, S. Yu. Sivoklokov, J. Sjölin, T. B. Sjursen, M. B. Skinner, H. P. Skottowe, P. Skubic, M. Slater, T. Slavicek, M. Slawinska, K. Sliwa, R. Slovak, V. Smakhtin, B. H. Smart, L. Smestad, S. Yu. Smirnov, Y. Smirnov, L. N. Smirnova, O. Smirnova, M. N. K. Smith, R. W. Smith, M. Smizanska, K. Smolek, A. A. Snesarev, G. Snidero, S. Snyder, R. Sobie, F. Socher, A. Soffer, D. A. Soh, G. Sokhrannyi, C. A. Solans Sanchez, M. Solar, E. Yu. Soldatov, U. Soldevila, A. A. Solodkov, A. Soloshenko, O. V. Solovyanov, V. Solovyev, P. Sommer, H. Son, H. Y. Song, A. Sood, A. Sopczak, V. Sopko, V. Sorin, D. Sosa, C. L. Sotiropoulou, R. Soualah, A. M. Soukharev, D. South, B. C. Sowden, S. Spagnolo, M. Spalla, M. Spangenberg, F. Spanò, D. Sperlich, F. Spettel, R. Spighi, G. Spigo, L. A. Spiller, M. Spousta, R. D. St. Denis, A. Stabile, S. Staerz, J. Stahlman, R. Stamen, S. Stamm, E. Stanecka, R. W. Stanek, C. Stanescu, M. Stanescu-Bellu, M. M. Stanitzki, S. Stapnes, E. A. Starchenko, G. H. Stark, J. Stark, P. Staroba, P. Starovoitov, R. Staszewski, P. Steinberg, B. Stelzer, H. J. Stelzer, O. Stelzer-Chilton, H. Stenzel, G. A. Stewart, J. A. Stillings, M. C. Stockton, M. Stoebe, G. Stoicea, P. Stolte, S. Stonjek, A. R. Stradling, A. Straessner, M. E. Stramaglia, J. Strandberg, S. Strandberg, A. Strandlie, M. Strauss, P. Strizenec, R. Ströhmer, D. M. Strom, R. Stroynowski, A. Strubig, S. A. Stucci, B. Stugu, N. A. Styles, D. Su, J. Su, R. Subramaniam, S. Suchek, Y. Sugaya, M. Suk, V. V. Sulin, S. Sultansoy, T. Sumida, S. Sun, X. Sun, J. E. Sundermann, K. Suruliz, G. Susinno, M. R. Sutton, S. Suzuki, M. Svatos, M. Swiatlowski, I. Sykora, T. Sykora, D. Ta, C. Taccini, K. Tackmann, J. Taenzer, A. Taffard, R. Tafirout, N. Taiblum, H. Takai, R. Takashima, H. Takeda, T. Takeshita, Y. Takubo, M. Talby, A. A. Talyshev, J. Y. C. Tam, K. G. Tan, J. Tanaka, R. Tanaka, S. Tanaka, B. B. Tannenwald, S. Tapia Araya, S. Tapprogge, S. Tarem, G. F. Tartarelli, P. Tas, M. Tasevsky, T. Tashiro, E. Tassi, A. Tavares Delgado, Y. Tayalati, A. C. Taylor, G. N. Taylor, P. T. E. Taylor, W. Taylor, F. A. Teischinger, P. Teixeira-Dias, K. K. Temming, D. Temple, H. Ten Kate, P. K. Teng, J. J. Teoh, F. Tepel, S. Terada, K. Terashi, J. Terron, S. Terzo, M. Testa, R. J. Teuscher, T. Theveneaux-Pelzer, J. P. Thomas, J. Thomas-Wilsker, E. N. Thompson, P. D. Thompson, R. J. Thompson, A. S. Thompson, L. A. Thomsen, E. Thomson, M. Thomson, M. J. Tibbetts, R. E. Ticse Torres, V. O. Tikhomirov, Yu. A. Tikhonov, S. Timoshenko, P. Tipton, S. Tisserant, K. Todome, T. Todorov, S. Todorova-Nova, J. Tojo, S. Tokár, K. Tokushuku, E. Tolley, L. Tomlinson, M. Tomoto, L. Tompkins, K. Toms, B. Tong, E. Torrence, H. Torres, E. Torró Pastor, J. Toth, F. Touchard, D. R. Tovey, T. Trefzger, L. Tremblet, A. Tricoli, I. M. Trigger, S. Trincaz-Duvoid, M. F. Tripiana, W. Trischuk, B. Trocmé, A. Trofymov, C. Troncon, M. Trottier-McDonald, M. Trovatelli, L. Truong, M. Trzebinski, A. Trzupek, J. C-L. Tseng, P. V. Tsiareshka, G. Tsipolitis, N. Tsirintanis, S. Tsiskaridze, V. Tsiskaridze, E. G. Tskhadadze, K. M. Tsui, I. I. Tsukerman, V. Tsulaia, S. Tsuno, D. Tsybychev, A. Tudorache, V. Tudorache, A. N. Tuna, S. A. Tupputi, S. Turchikhin, D. Turecek, D. Turgeman, R. Turra, A. J. Turvey, P. M. Tuts, M. Tyndel, G. Ucchielli, I. Ueda, R. Ueno, M. Ughetto, F. Ukegawa, G. Unal, A. Undrus, G. Unel, F. C. Ungaro, Y. Unno, C. Unverdorben, J. Urban, P. Urquijo, P. Urrejola, G. Usai, A. Usanova, L. Vacavant, V. Vacek, B. Vachon, C. Valderanis, E. Valdes Santurio, N. Valencic, S. Valentinetti, A. Valero, L. Valery, S. Valkar, S. Vallecorsa, J. A. Valls Ferrer, W. Van Den Wollenberg, P. C. Van Der Deijl, R. van der Geer, H. van der Graaf, N. van Eldik, P. van Gemmeren, J. Van Nieuwkoop, I. van Vulpen, M. C. van Woerden, M. Vanadia, W. Vandelli, R. Vanguri, A. Vaniachine, P. Vankov, G. Vardanyan, R. Vari, E. W. Varnes, T. Varol, D. Varouchas, A. Vartapetian, K. E. Varvell, J. G. Vasquez, F. Vazeille, T. Vazquez Schroeder, J. Veatch, L. M. Veloce, F. Veloso, S. Veneziano, A. Ventura, M. Venturi, N. Venturi, A. Venturini, V. Vercesi, M. Verducci, W. Verkerke, J. C. Vermeulen, A. Vest, M. C. Vetterli, O. Viazlo, I. Vichou, T. Vickey, O. E. Vickey Boeriu, G. H. A. Viehhauser, S. Viel, L. Vigani, R. Vigne, M. Villa, M. Villaplana Perez, E. Vilucchi, M. G. Vincter, V. B. Vinogradov, C. Vittori, I. Vivarelli, S. Vlachos, M. Vlasak, M. Vogel, P. Vokac, G. Volpi, M. Volpi, H. von der Schmitt, E. von Toerne, V. Vorobel, K. Vorobev, M. Vos, R. Voss, J. H. Vossebeld, N. Vranjes, M. Vranjes Milosavljevic, V. Vrba, M. Vreeswijk, R. Vuillermet, I. Vukotic, Z. Vykydal, P. Wagner, W. Wagner, H. Wahlberg, S. Wahrmund, J. Wakabayashi, J. Walder, R. Walker, W. Walkowiak, V. Wallangen, C. Wang, C. Wang, F. Wang, H. Wang, H. Wang, J. Wang, J. Wang, K. Wang, R. Wang, S. M. Wang, T. Wang, T. Wang, X. Wang, C. Wanotayaroj, A. Warburton, C. P. Ward, D. R. Wardrope, A. Washbrook, P. M. Watkins, A. T. Watson, I. J. Watson, M. F. Watson, G. Watts, S. Watts, B. M. Waugh, S. Webb, M. S. Weber, S. W. Weber, J. S. Webster, A. R. Weidberg, B. Weinert, J. Weingarten, C. Weiser, H. Weits, P. S. Wells, T. Wenaus, T. Wengler, S. Wenig, N. Wermes, M. Werner, P. Werner, M. Wessels, J. Wetter, K. Whalen, N. L. Whallon, A. M. Wharton, A. White, M. J. White, R. White, S. White, D. Whiteson, F. J. Wickens, W. Wiedenmann, M. Wielers, P. Wienemann, C. Wiglesworth, L. A. M. Wiik-Fuchs, A. Wildauer, F. Wilk, H. G. Wilkens, H. H. Williams, S. Williams, C. Willis, S. Willocq, J. A. Wilson, I. Wingerter-Seez, F. Winklmeier, O. J. Winston, B. T. Winter, M. Wittgen, J. Wittkowski, S. J. Wollstadt, M. W. Wolter, H. Wolters, B. K. Wosiek, J. Wotschack, M. J. Woudstra, K. W. Wozniak, M. Wu, M. Wu, S. L. Wu, X. Wu, Y. Wu, T. R. Wyatt, B. M. Wynne, S. Xella, D. Xu, L. Xu, B. Yabsley, S. Yacoob, R. Yakabe, D. Yamaguchi, Y. Yamaguchi, A. Yamamoto, S. Yamamoto, T. Yamanaka, K. Yamauchi, Y. Yamazaki, Z. Yan, H. Yang, H. Yang, Y. Yang, Z. Yang, W-M. Yao, Y. C. Yap, Y. Yasu, E. Yatsenko, K. H. Yau Wong, J. Ye, S. Ye, I. Yeletskikh, A. L. Yen, E. Yildirim, K. Yorita, R. Yoshida, K. Yoshihara, C. Young, C. J. S. Young, S. Youssef, D. R. Yu, J. Yu, J. M. Yu, J. Yu, L. Yuan, S. P. Y. Yuen, I. Yusuff, B. Zabinski, R. Zaidan, A. M. Zaitsev, N. Zakharchuk, J. Zalieckas, A. Zaman, S. Zambito, L. Zanello, D. Zanzi, C. Zeitnitz, M. Zeman, A. Zemla, J. C. Zeng, Q. Zeng, K. Zengel, O. Zenin, T. Ženiš, D. Zerwas, D. Zhang, F. Zhang, G. Zhang, H. Zhang, J. Zhang, L. Zhang, R. Zhang, R. Zhang, X. Zhang, Z. Zhang, X. Zhao, Y. Zhao, Z. Zhao, A. Zhemchugov, J. Zhong, B. Zhou, C. Zhou, L. Zhou, L. Zhou, M. Zhou, N. Zhou, C. G. Zhu, H. Zhu, J. Zhu, Y. Zhu, X. Zhuang, K. Zhukov, A. Zibell, D. Zieminska, N. I. Zimine, C. Zimmermann, S. Zimmermann, Z. Zinonos, M. Zinser, M. Ziolkowski, L. Živković, G. Zobernig, A. Zoccoli, M. zur Nedden, G. Zurzolo, L. Zwalinski, ATLAS Collaboration

**Affiliations:** 1 Department of Physics, University of Adelaide, Adelaide, Australia; 2 Physics Department, SUNY Albany, Albany, NY USA; 3 Department of Physics, University of Alberta, Edmonton, AB Canada; 4 Department of Physics, Ankara University, Ankara, Turkey; 5 Istanbul Aydin University, Istanbul, Turkey; 6 Division of Physics, TOBB University of Economics and Technology, Ankara, Turkey; 7 LAPP, CNRS/IN2P3 and Université Savoie Mont Blanc, Annecy-le-Vieux, France; 8 High Energy Physics Division, Argonne National Laboratory, Argonne, IL USA; 9 Department of Physics, University of Arizona, Tucson, AZ USA; 10 Department of Physics, The University of Texas at Arlington, Arlington, TX USA; 11 Physics Department, University of Athens, Athens, Greece; 12 Physics Department, National Technical University of Athens, Zografou, Greece; 13 Institute of Physics, Azerbaijan Academy of Sciences, Baku, Azerbaijan; 14 Institut de Física d’Altes Energies (IFAE), The Barcelona Institute of Science and Technology, Barcelona, Spain; 15 Institute of Physics, University of Belgrade, Belgrade, Serbia; 16 Department for Physics and Technology, University of Bergen, Bergen, Norway; 17 Physics Division, Lawrence Berkeley National Laboratory and University of California, Berkeley, CA USA; 18 Department of Physics, Humboldt University, Berlin, Germany; 19 Albert Einstein Center for Fundamental Physics and Laboratory for High Energy Physics, University of Bern, Bern, Switzerland; 20 School of Physics and Astronomy, University of Birmingham, Birmingham, UK; 21 Department of Physics, Bogazici University, Istanbul, Turkey; 22 Department of Physics Engineering, Gaziantep University, Gaziantep, Turkey; 23 Faculty of Engineering and Natural Sciences, Istanbul Bilgi University, Istanbul, Turkey; 24 Faculty of Engineering and Natural Sciences, Bahcesehir University, Istanbul, Turkey; 25 Centro de Investigaciones, Universidad Antonio Narino, Bogota, Colombia; 26 INFN Sezione di Bologna, Bologna, Italy; 27 Dipartimento di Fisica e Astronomia, Università di Bologna, Bologna, Italy; 28 Physikalisches Institut, University of Bonn, Bonn, Germany; 29 Department of Physics, Boston University, Boston, MA USA; 30 Department of Physics, Brandeis University, Waltham, MA USA; 31 Universidade Federal do Rio De Janeiro COPPE/EE/IF, Rio de Janeiro, Brazil; 32 Electrical Circuits Department, Federal University of Juiz de Fora (UFJF), Juiz de Fora, Brazil; 33 Federal University of Sao Joao del Rei (UFSJ), Sao Joao del Rei, Brazil; 34 Instituto de Fisica, Universidade de Sao Paulo, São Paulo, Brazil; 35 Physics Department, Brookhaven National Laboratory, Upton, NY USA; 36 Transilvania University of Brasov, Brasov, Romania; 37 National Institute of Physics and Nuclear Engineering, Bucharest, Romania; 38 Physics Department, National Institute for Research and Development of Isotopic and Molecular Technologies, Cluj Napoca, Romania; 39 University Politehnica Bucharest, Bucharest, Romania; 40 West University in Timisoara, Timisoara, Romania; 41 Departamento de Física, Universidad de Buenos Aires, Buenos Aires, Argentina; 42 Cavendish Laboratory, University of Cambridge, Cambridge, UK; 43 Department of Physics, Carleton University, Ottawa, ON Canada; 44 CERN, Geneva, Switzerland; 45 Enrico Fermi Institute, University of Chicago, Chicago, IL USA; 46 Departamento de Física, Pontificia Universidad Católica de Chile, Santiago, Chile; 47 Departamento de Física, Universidad Técnica Federico Santa María, Valparaíso, Chile; 48 Institute of High Energy Physics, Chinese Academy of Sciences, Beijing, China; 49 Department of Modern Physics, University of Science and Technology of China, Hefei, Anhui China; 50 Department of Physics, Nanjing University, Nanjing, Jiangsu China; 51 School of Physics, Shandong University, Jinan, Shandong China; 52 Shanghai Key Laboratory for Particle Physics and Cosmology, Department of Physics and Astronomy, Shanghai Jiao Tong University, (also affiliated with PKU-CHEP), Shanghai, China; 53 Physics Department, Tsinghua University, Beijing, 100084 China; 54 Laboratoire de Physique Corpusculaire, Clermont Université and Université Blaise Pascal and CNRS/IN2P3, Clermont-Ferrand, France; 55 Nevis Laboratory, Columbia University, Irvington, NY USA; 56 Niels Bohr Institute, University of Copenhagen, Kobenhavn, Denmark; 57 INFN Gruppo Collegato di Cosenza, Laboratori Nazionali di Frascati, Frascati, Italy; 58 Dipartimento di Fisica, Università della Calabria, Rende, Italy; 59 Faculty of Physics and Applied Computer Science, AGH University of Science and Technology, Kraków, Poland; 60 Marian Smoluchowski Institute of Physics, Jagiellonian University, Kraków, Poland; 61 Institute of Nuclear Physics, Polish Academy of Sciences, Kraków, Poland; 62 Physics Department, Southern Methodist University, Dallas, TX USA; 63 Physics Department, University of Texas at Dallas, Richardson, TX USA; 64 DESY, Hamburg and Zeuthen, Germany; 65 Institut für Experimentelle Physik IV, Technische Universität Dortmund, Dortmund, Germany; 66 Institut für Kern- und Teilchenphysik, Technische Universität Dresden, Dresden, Germany; 67 Department of Physics, Duke University, Durham, NC USA; 68 SUPA-School of Physics and Astronomy, University of Edinburgh, Edinburgh, UK; 69 INFN Laboratori Nazionali di Frascati, Frascati, Italy; 70 Fakultät für Mathematik und Physik, Albert-Ludwigs-Universität, Freiburg, Germany; 71 Section de Physique, Université de Genève, Geneva, Switzerland; 72 INFN Sezione di Genova, Genoa, Italy; 73 Dipartimento di Fisica, Università di Genova, Genoa, Italy; 74 E. Andronikashvili Institute of Physics, Iv. Javakhishvili Tbilisi State University, Tbilisi, Georgia; 75 High Energy Physics Institute, Tbilisi State University, Tbilisi, Georgia; 76 II Physikalisches Institut, Justus-Liebig-Universität Giessen, Giessen, Germany; 77 SUPA-School of Physics and Astronomy, University of Glasgow, Glasgow, UK; 78 II Physikalisches Institut, Georg-August-Universität, Göttingen, Germany; 79 Laboratoire de Physique Subatomique et de Cosmologie, Université Grenoble-Alpes, CNRS/IN2P3, Grenoble, France; 80 Department of Physics, Hampton University, Hampton, VA USA; 81 Laboratory for Particle Physics and Cosmology, Harvard University, Cambridge, MA USA; 82 Kirchhoff-Institut für Physik, Ruprecht-Karls-Universität Heidelberg, Heidelberg, Germany; 83 Physikalisches Institut, Ruprecht-Karls-Universität Heidelberg, Heidelberg, Germany; 84 ZITI Institut für technische Informatik, Ruprecht-Karls-Universität Heidelberg, Mannheim, Germany; 85 Faculty of Applied Information Science, Hiroshima Institute of Technology, Hiroshima, Japan; 86 Department of Physics, The Chinese University of Hong Kong, Shatin, NT Hong Kong; 87 Department of Physics, The University of Hong Kong, Hong Kong, China; 88 Department of Physics, The Hong Kong University of Science and Technology, Clear Water Bay, Kowloon, Hong Kong China; 89 Department of Physics, Indiana University, Bloomington, IN USA; 90 Institut für Astro- und Teilchenphysik, Leopold-Franzens-Universität, Innsbruck, Austria; 91 University of Iowa, Iowa City, IA USA; 92 Department of Physics and Astronomy, Iowa State University, Ames, IA USA; 93 Joint Institute for Nuclear Research, JINR Dubna, Dubna, Russia; 94 KEK, High Energy Accelerator Research Organization, Tsukuba, Japan; 95 Graduate School of Science, Kobe University, Kobe, Japan; 96 Faculty of Science, Kyoto University, Kyoto, Japan; 97 Kyoto University of Education, Kyoto, Japan; 98 Department of Physics, Kyushu University, Fukuoka, Japan; 99 Instituto de Física La Plata, Universidad Nacional de La Plata and CONICET, La Plata, Argentina; 100 Physics Department, Lancaster University, Lancaster, UK; 101 INFN Sezione di Lecce, Lecce, Italy; 102 Dipartimento di Matematica e Fisica, Università del Salento, Lecce, Italy; 103 Oliver Lodge Laboratory, University of Liverpool, Liverpool, UK; 104 Department of Physics, Jožef Stefan Institute and University of Ljubljana, Ljubljana, Slovenia; 105 School of Physics and Astronomy, Queen Mary University of London, London, UK; 106 Department of Physics, Royal Holloway University of London, Surrey, UK; 107 Department of Physics and Astronomy, University College London, London, UK; 108 Louisiana Tech University, Ruston, LA USA; 109 Laboratoire de Physique Nucléaire et de Hautes Energies, UPMC and Université Paris-Diderot and CNRS/IN2P3, Paris, France; 110 Fysiska Institutionen, Lunds Universitet, Lund, Sweden; 111 Departamento de Fisica Teorica C-15, Universidad Autonoma de Madrid, Madrid, Spain; 112 Institut für Physik, Universität Mainz, Mainz, Germany; 113 School of Physics and Astronomy, University of Manchester, Manchester, UK; 114 CPPM, Aix-Marseille Université and CNRS/IN2P3, Marseille, France; 115 Department of Physics, University of Massachusetts, Amherst, MA USA; 116 Department of Physics, McGill University, Montreal, QC Canada; 117 School of Physics, University of Melbourne, Melbourne, Victoria Australia; 118 Department of Physics, The University of Michigan, Ann Arbor, MI USA; 119 Department of Physics and Astronomy, Michigan State University, East Lansing, MI USA; 120 INFN Sezione di Milano, Milan, Italy; 121 Dipartimento di Fisica, Università di Milano, Milan, Italy; 122 B.I. Stepanov Institute of Physics, National Academy of Sciences of Belarus, Minsk, Republic of Belarus; 123 National Scientific and Educational Centre for Particle and High Energy Physics, Minsk, Republic of Belarus; 124 Group of Particle Physics, University of Montreal, Montreal, QC Canada; 125 P.N. Lebedev Physical Institute of the Russian, Academy of Sciences, Moscow, Russia; 126 Institute for Theoretical and Experimental Physics (ITEP), Moscow, Russia; 127 National Research Nuclear University MEPhI, Moscow, Russia; 128 D.V. Skobeltsyn Institute of Nuclear Physics, M.V. Lomonosov Moscow State University, Moscow, Russia; 129 Fakultät für Physik, Ludwig-Maximilians-Universität München, Munich, Germany; 130 Max-Planck-Institut für Physik (Werner-Heisenberg-Institut), Munich, Germany; 131 Nagasaki Institute of Applied Science, Nagasaki, Japan; 132 Graduate School of Science and Kobayashi-Maskawa Institute, Nagoya University, Nagoya, Japan; 133 INFN Sezione di Napoli, Naples, Italy; 134 Dipartimento di Fisica, Università di Napoli, Naples, Italy; 135 Department of Physics and Astronomy, University of New Mexico, Albuquerque, NM USA; 136 Institute for Mathematics, Astrophysics and Particle Physics, Radboud University Nijmegen/Nikhef, Nijmegen, The Netherlands; 137 Nikhef National Institute for Subatomic Physics and University of Amsterdam, Amsterdam, The Netherlands; 138 Department of Physics, Northern Illinois University, DeKalb, IL USA; 139 Budker Institute of Nuclear Physics, SB RAS, Novosibirsk, Russia; 140 Department of Physics, New York University, New York, NY USA; 141 Ohio State University, Columbus, OH USA; 142 Faculty of Science, Okayama University, Okayama, Japan; 143 Homer L. Dodge Department of Physics and Astronomy, University of Oklahoma, Norman, OK USA; 144 Department of Physics, Oklahoma State University, Stillwater, OK USA; 145 Palacký University, RCPTM, Olomouc, Czech Republic; 146 Center for High Energy Physics, University of Oregon, Eugene, OR USA; 147 LAL, Univ. Paris-Sud, CNRS/IN2P3, Université Paris-Saclay, Orsay, France; 148 Graduate School of Science, Osaka University, Osaka, Japan; 149 Department of Physics, University of Oslo, Oslo, Norway; 150 Department of Physics, Oxford University, Oxford, UK; 151 INFN Sezione di Pavia, Pavia, Italy; 152 Dipartimento di Fisica, Università di Pavia, Pavia, Italy; 153 Department of Physics, University of Pennsylvania, Philadelphia, PA USA; 154 National Research Centre “Kurchatov Institute” B.P.Konstantinov Petersburg Nuclear Physics Institute, St. Petersburg, Russia; 155 INFN Sezione di Pisa, Pisa, Italy; 156 Dipartimento di Fisica E. Fermi, Università di Pisa, Pisa, Italy; 157 Department of Physics and Astronomy, University of Pittsburgh, Pittsburgh, PA USA; 158 Laboratório de Instrumentação e Física Experimental de Partículas-LIP, Lisbon, Portugal; 159 Faculdade de Ciências, Universidade de Lisboa, Lisbon, Portugal; 160 Department of Physics, University of Coimbra, Coimbra, Portugal; 161 Centro de Física Nuclear da Universidade de Lisboa, Lisbon, Portugal; 162 Departamento de Fisica, Universidade do Minho, Braga, Portugal; 163 Departamento de Fisica Teorica y del Cosmos and CAFPE, Universidad de Granada, Granada, Spain; 164 Dep Fisica and CEFITEC of Faculdade de Ciencias e Tecnologia, Universidade Nova de Lisboa, Caparica, Portugal; 165 Institute of Physics, Academy of Sciences of the Czech Republic, Prague, Czech Republic; 166 Czech Technical University in Prague, Prague, Czech Republic; 167 Faculty of Mathematics and Physics, Charles University in Prague, Prague, Czech Republic; 168 State Research Center Institute for High Energy Physics (Protvino), NRC KI, Protvino, Russia; 169 Particle Physics Department, Rutherford Appleton Laboratory, Didcot, UK; 170 INFN Sezione di Roma, Rome, Italy; 171 Dipartimento di Fisica, Sapienza Università di Roma, Rome, Italy; 172 INFN Sezione di Roma Tor Vergata, Rome, Italy; 173 Dipartimento di Fisica, Università di Roma Tor Vergata, Rome, Italy; 174 INFN Sezione di Roma Tre, Rome, Italy; 175 Dipartimento di Matematica e Fisica, Università Roma Tre, Rome, Italy; 176 Faculté des Sciences Ain Chock, Réseau Universitaire de Physique des Hautes Energies-Université Hassan II, Casablanca, Morocco; 177 Centre National de l’Energie des Sciences Techniques Nucleaires, Rabat, Morocco; 178 Faculté des Sciences Semlalia, Université Cadi Ayyad, LPHEA-Marrakech, Marrakech, Morocco; 179 Faculté des Sciences, Université Mohamed Premier and LPTPM, Oujda, Morocco; 180 Faculté des Sciences, Université Mohammed V, Rabat, Morocco; 181 DSM/IRFU (Institut de Recherches sur les Lois Fondamentales de l’Univers), CEA Saclay (Commissariat à l’Energie Atomique et aux Energies Alternatives), Gif-sur-Yvette, France; 182 Santa Cruz Institute for Particle Physics, University of California Santa Cruz, Santa Cruz, CA USA; 183 Department of Physics, University of Washington, Seattle, WA USA; 184 Department of Physics and Astronomy, University of Sheffield, Sheffield, UK; 185 Department of Physics, Shinshu University, Nagano, Japan; 186 Fachbereich Physik, Universität Siegen, Siegen, Germany; 187 Department of Physics, Simon Fraser University, Burnaby, BC Canada; 188 SLAC National Accelerator Laboratory, Stanford, CA USA; 189 Faculty of Mathematics, Physics and Informatics, Comenius University, Bratislava, Slovak Republic; 190 Department of Subnuclear Physics, Institute of Experimental Physics of the Slovak Academy of Sciences, Kosice, Slovak Republic; 191 Department of Physics, University of Cape Town, Cape Town, South Africa; 192 Department of Physics, University of Johannesburg, Johannesburg, South Africa; 193 School of Physics, University of the Witwatersrand, Johannesburg, South Africa; 194 Department of Physics, Stockholm University, Stockholm, Sweden; 195 The Oskar Klein Centre, Stockholm, Sweden; 196 Physics Department, Royal Institute of Technology, Stockholm, Sweden; 197 Departments of Physics and Astronomy and Chemistry, Stony Brook University, Stony Brook, NY USA; 198 Department of Physics and Astronomy, University of Sussex, Brighton, UK; 199 School of Physics, University of Sydney, Sydney, Australia; 200 Institute of Physics, Academia Sinica, Taipei, Taiwan; 201 Department of Physics, Technion: Israel Institute of Technology, Haifa, Israel; 202 Raymond and Beverly Sackler School of Physics and Astronomy, Tel Aviv University, Tel Aviv, Israel; 203 Department of Physics, Aristotle University of Thessaloniki, Thessaloníki, Greece; 204 International Center for Elementary Particle Physics and Department of Physics, The University of Tokyo, Tokyo, Japan; 205 Graduate School of Science and Technology, Tokyo Metropolitan University, Tokyo, Japan; 206 Department of Physics, Tokyo Institute of Technology, Tokyo, Japan; 207 Department of Physics, University of Toronto, Toronto, ON Canada; 208 TRIUMF, Vancouver, BC Canada; 209 Department of Physics and Astronomy, York University, Toronto, ON Canada; 210 Faculty of Pure and Applied Sciences, and Center for Integrated Research in Fundamental Science and Engineering, University of Tsukuba, Tsukuba, Japan; 211 Department of Physics and Astronomy, Tufts University, Medford, MA USA; 212 Department of Physics and Astronomy, University of California Irvine, Irvine, CA USA; 213 INFN Gruppo Collegato di Udine, Sezione di Trieste, Udine, Italy; 214 ICTP, Trieste, Italy; 215 Dipartimento di Chimica Fisica e Ambiente, Università di Udine, Udine, Italy; 216 Department of Physics and Astronomy, University of Uppsala, Uppsala, Sweden; 217 Department of Physics, University of Illinois, Urbana, IL USA; 218 Instituto de Física Corpuscular (IFIC) and Departamento de Física Atómica, Molecular y Nuclear and Departamento de Ingeniería Electrónica and Instituto de Microelectrónica de Barcelona (IMB-CNM), University of Valencia and CSIC, Valencia, Spain; 219 Department of Physics, University of British Columbia, Vancouver, BC Canada; 220 Department of Physics and Astronomy, University of Victoria, Victoria, BC Canada; 221 Department of Physics, University of Warwick, Coventry, UK; 222 Waseda University, Tokyo, Japan; 223 Department of Particle Physics, The Weizmann Institute of Science, Rehovot, Israel; 224 Department of Physics, University of Wisconsin, Madison, WI USA; 225 Fakultät für Physik und Astronomie, Julius-Maximilians-Universität, Würzburg, Germany; 226 Fakultät für Mathematik und Naturwissenschaften, Fachgruppe Physik, Bergische Universität Wuppertal, Wuppertal, Germany; 227 Department of Physics, Yale University, New Haven, CT USA; 228 Yerevan Physics Institute, Yerevan, Armenia; 229 Centre de Calcul de l’Institut National de Physique Nucléaire et de Physique des Particules (IN2P3), Villeurbanne, France; 230 CERN, 1211 Geneva 23, Switzerland

## Abstract

This paper presents measurements of distributions of charged particles which are produced in proton–proton collisions at a centre-of-mass energy of $\sqrt{s} = 8 \mathrm {\, TeV}$ and recorded by the ATLAS detector at the LHC. A special dataset recorded in 2012 with a small number of interactions per beam crossing (below 0.004) and corresponding to an integrated luminosity of 160 $\upmu \mathrm{b}^{-1}$ was used. A minimum-bias trigger was utilised to select a data sample of more than 9 million collision events. The multiplicity, pseudorapidity, and transverse momentum distributions of charged particles are shown in different regions of kinematics and charged-particle multiplicity, including measurements of final states at high multiplicity. The results are corrected for detector effects and are compared to the predictions of various Monte Carlo event generator models which simulate the full hadronic final state.

## Introduction

Measurements of charged-particle spectra probe strong interactions at low momentum transfers. Such measurements have been made in lower-energy $e^+e^-$, *ep* and hadron collisions [1–11] and at the CERN Large Hadron Collider (LHC) [12–23]. This paper presents measurements of multiplicity distributions, as well as transverse momentum and pseudorapidity spectra, for primary charged particles produced in *pp* collisions recorded by the ATLAS experiment [24] at the LHC at $8 \mathrm {\, TeV}$ centre-of-mass energy.

Although a description of low-energy processes within a perturbative framework is not possible, predictions can be made with phenomenological models inspired by quantum chromodynamics (QCD). Data are used to constrain such models and gain further insight into the particle dynamics of the low transverse momentum regime. Furthermore, low-$p_{\text {T}}$ processes, arising from pile-up in which there is more than one interaction per beam crossing, may also affect the topologies of events involving an interaction with a high $p_{\text {T}}$ scale. An understanding of soft QCD processes is therefore important both in its own right and as a means of reducing systematic uncertainties in measurements of high-$p_{\text {T}}$ phenomena.

The measurements presented in this paper use a methodology similar to that used at lower centre-of-mass energies at ATLAS [18]. Events were selected from data taken in 2012 with a trigger overlapping with the acceptance of the tracking volume. This corresponds to a minimum-bias dataset based on inelastic *pp* interactions. The term *minimum bias* is taken to refer to trigger and event selections which are as unrestrictive as possible for the *pp*-induced final states. The integrated luminosity of the data sample under study is 160 $\upmu \mathrm{b}^{-1}$. Owing to improvements in understanding the material inside and around the ATLAS inner detector (ID), the uncertainties in the measured spectra are reduced by as much as 30–50 % compared to the analogous measurements at $7 \mathrm {\, TeV}$ centre-of-mass energy [18].

The following distributions are measured:

$$\begin{aligned}&{1}/{N_{\mathrm {ev}}} \cdot {\mathrm {d} N_{\mathrm {ch}}}/{\mathrm {d} \eta }\ , \quad {1}/({2 \pi p_{\text {T}} {N_{\mathrm {ev}}}}) \cdot {\mathrm {d}^2 N_{\mathrm {ch}}}/({\mathrm {d} \eta \, \mathrm {d} p_{\text {T}}})\ , \\&{1}/{N_{\mathrm {ev}}} \cdot {{\mathrm {d} N_{\mathrm {ev}}}/{\mathrm {d} n_{\mathrm {ch}}}}\ , \quad \mathrm {and} \quad \langle p_{\text {T}} \rangle \ \mathrm {vs} \ n_{\mathrm {ch}}\ . \end{aligned}$$

Here, $\eta$ is the particle’s pseudorapidity,^1^ $p_{\text {T}}$ is the component of the charged-particle momentum which is transverse to the beam direction,^2^ $n_{\mathrm {ch}}$ is the number of primary charged particles in an event, $N_{\mathrm {ev}}$ is the event yield for a given event selection, and $N_{\mathrm {ch}}$ is the total number of primary charged particles in all selected events in the data sample. A primary charged particle is defined as a charged particle with a mean lifetime $\tau > 300\,\mathrm{ps}$, which is either directly produced in *pp* interactions or from decays of directly produced particles with $\tau < 30\, \mathrm{ps}$; particles produced from decays of particles with $\tau > 30\, \mathrm{ps}$ are considered as secondary particles and are thus excluded. Primary charged particles are furthermore required to satisfy the kinematic selection criteria of $|\eta | < 2.5$ and either $p_{\text {T}} > 100$ or $500 \mathrm {\, MeV}$.

In order to make a more complete study of particle properties in minimum-bias events, results are given for different multiplicity and kinematic selections (termed *phase spaces*). The most inclusive phase spaces correspond to events with a minimum multiplicity $n_{\mathrm {ch}} \ge 2$ or 1 and contain primary charged particles possessing a minimum transverse momentum $p_{\text {T}} > 100$ or $500 \mathrm {\, MeV}$, respectively. Primary-charged-particle spectra are also shown for higher-multiplicity events ($p_{\text {T}} > 500 \mathrm {\, MeV}$, $n_{\mathrm {ch}} \ge 6,20$ and 50) of which the latter two event types have hitherto not been measured by ATLAS. Finally, the average primary-charged-particle densities at central pseudorapidity are compared to existing measurements at different centre-of-mass energies.

## ATLAS detector

The ATLAS detector covers almost the whole solid angle around the collision point with layers of tracking detectors, calorimeters and muon chambers. The tracking modules and the trigger system are of most relevance for the presented measurements.

The inner detector has full coverage in $\phi$ and covers the pseudorapidity range $|\eta | < 2.5$. It comprises a silicon pixel detector (Pixel), a silicon microstrip detector (SCT) and a transition radiation tracker (TRT). These detectors cover a sensitive radial distance from the interaction point of 50.5–150  mm, 299–560  mm and 563–1066  mm, respectively, and are immersed in a 2 T axial magnetic field provided by a solenoid. The inner-detector barrel (end-cap) parts consist of 3 ($2\times 3$) Pixel layers, 4 ($2 \times 9$) double-layers of single-sided silicon microstrips with a 40 mrad stereo angle, and 73 ($2 \times 160$) layers of TRT straws. Typical position resolutions are 10, 17 and 130 $\upmu$m for the *r*–$\phi$ co-ordinate and, in the case of the Pixel and SCT, 115 and 580 $\upmu$m for the second measured co-ordinate. A track from a primary charged particle traversing the barrel detector would typically have 11 silicon hits^3^ (3 pixel clusters and 8 strip clusters) and more than 30 TRT straw hits.

The ATLAS detector has a three-level trigger system: Level 1 (L1), Level 2 (L2) and Event Filter (EF). For the presented measurements, the trigger relies on the L1 signals from the minimum-bias trigger scintillators (MBTS). The MBTS are positioned at each end of the detector in front of the liquid-argon end-cap calorimeter cryostats at $z = \pm 3.56\,\mathrm{m}$. They are segmented into eight sectors in azimuth and two rings in pseudorapidity and cover the range $2.08< |\eta | < 3.75$. The MBTS triggers are configured to require at least one or two hits above threshold from either side of the detector.^4^

## Monte Carlo simulation

The following Monte Carlo (MC) models of inclusive hadron–hadron interactions were used to generate event samples. These models employ different settings of model parameters (referred to as *tunes*) which were optimised to reproduce existing experimental data.

- Pythia 8 [25] and Pythia 6 [26]. In these models, the total inelastic cross-section is separated into non-diffractive (ND) processes, dominated by *t*-channel gluon exchange, and diffractive processes where a colour-singlet object is exchanged. Multiple parton–parton interactions (MPI) contribute to multiplicity fluctuations and are simulated as part of the ND processes. The diffractive processes consist of single-diffractive dissociation (SD) and double-diffractive dissociation (DD). Pythia 8 is used with the A2 [27] and Monash [28] tunes. The A2 tune was performed on minimum-bias and underlying-event data, utilising the MSTW2008 LO [29] parton distribution function (PDF). The Monash tune was made using a re-analysis of fragmentation-sensitive measurements with $e^+e^-$ collisions, combined with minimum-bias and underlying-event tuning for hadron–hadron data, utilising the NNPDF23LO PDF. Pythia 6 employs the AMBT2B [30] tune with the CTEQ6L1 [31] PDF. The AMBT2B tune was evaluated using jet and minimum-bias data.
- Epos  [32]. This model implements a parton-based Gribov–Regge theory [33], which is an effective QCD-inspired field theory describing hard and soft scattering simultaneously. Epos has been primarily designed for Pb+Pb interactions and cosmic air showers. The LHC tune [34] is used here, which modifies the modelling of radial flow to be more applicable for small-volume, high-density regions, as are found in *pp* interactions.
- Qgsjet-II  [35] using the default tune. This model provides a phenomenological treatment of hadronic and nuclear interactions within a Reggeon field theory framework, and includes soft and semi-hard parton processes within the “semi-hard pomeron” approach. Qgsjet-II was also developed for the simulation of cosmic rays. Qgsjet-II and Epos calculations do not rely on the standard PDFs as used in the Pythia generators.

The Pythia 8 A2, Pythia 6 AMBT2B and Epos LHC models were used to generate event samples which were processed by the Geant 4-based [36] ATLAS simulation framework [37]. The simulation also takes into account inactive and inefficient regions of the ATLAS detector. The resulting datasets were used to derive corrections for detector effects and to evaluate systematic uncertainties.

Comparisons to the data corrected to particle level are made with generated events using the Pythia 8 A2 and Monash tunes, the Epos LHC tune, and the default Qgsjet-II tune. These comparisons are shown in Sect. 10.

## Event selection

A dedicated LHC *pp* run was used for which the average number of *pp* interactions per bunch crossing, $\langle \mu \rangle$, was low ($0.0028< \mu < 0.004$). The maximum instantaneous luminosity was approximately $1.8\times 10^{28}\,\mathrm{cm}^{-2}\,\mathrm{s}^{-1}$. Events were selected for which all subcomponents of the ID were operational and the solenoid magnet was on. Only events from colliding proton bunches in which the MBTS trigger recorded one or more modules above threshold on either side were considered. The MBTS trigger efficiency is described in detail in Sect. 7.1.

The following event selection criteria were applied:

- A primary vertex with at least two associated tracks constrained to the luminous *z*-region of the measured beam position (termed *beam spot*) was required. The tracks were required to possess $p_{\text {T}} > 100 \mathrm {\, MeV}$ and their transverse distance of closest approach to the beam spot ($d_\mathrm {0}^{\mathrm {BS}}$) was restricted such that $|d_\mathrm {0}^{\mathrm {BS}} | < 4\,\mathrm{mm}$.
- Events were rejected if they had at least one additional vertex with four or more associated tracks. Following this selection, the estimated fraction of remaining pile-up events with more than one *pp* interaction, based on $\langle \mu \rangle$, was about 0.002 %. Events containing additional vertices with less than four tracks are dominated by split vertices, where the vertex reconstruction algorithm wrongly reconstructs two vertices from tracks which actually originate from a single vertex [38], and by secondary interactions being reconstructed as another primary vertex. The fraction of events with split vertices or secondary interactions which are rejected by this criterion was estimated from simulation to be 0.01 %, which is negligible and therefore ignored.
- Depending on the phase space under study, additional selections were made on track multiplicity given the required minimum transverse momentum possessed by a track. A minimum number of selected tracks $n_{\mathrm {sel}} \ge 2$ with transverse momentum $p_{\text {T}} > 100 \mathrm {\, MeV}$, or $n_{\mathrm {sel}} \ge 1$ with $p_{\text {T}} > 500 \mathrm {\, MeV}$, which satisfy the constraints given in Sect. 5, was required.

Following the application of the above selections, the event yield is $9.2 \times 10^6$ for the most inclusive phase space at $n_{\mathrm {sel}} \ge 2$ and $p_{\text {T}} > 100 \mathrm {\, MeV}$. The phase space with the lowest number of events ($\sim \!6.4 \times 10^4$) corresponds to $n_{\mathrm {sel}} \ge 50$ and $p_{\text {T}} > 500 \mathrm {\, MeV}$.

## Track reconstruction and selection

Tracks were reconstructed using two approaches as in previous studies at $\sqrt{s} = 7 \mathrm {\, TeV}$ [18]. Firstly, an *inside-out* algorithm, starting the pattern recognition from clusters in the Pixel detector, was employed. An additional algorithm with relaxed requirements on the number of silicon hits was employed to reconstruct low-momentum tracks from hits which were unused in the first approach. This latter method increases the overall efficiency of finding low-$p_{\text {T}}$ tracks (mostly $100< p_{\text {T}} < 400 \mathrm {\, MeV}$) by up to a factor of two.

**Fig. 1:**
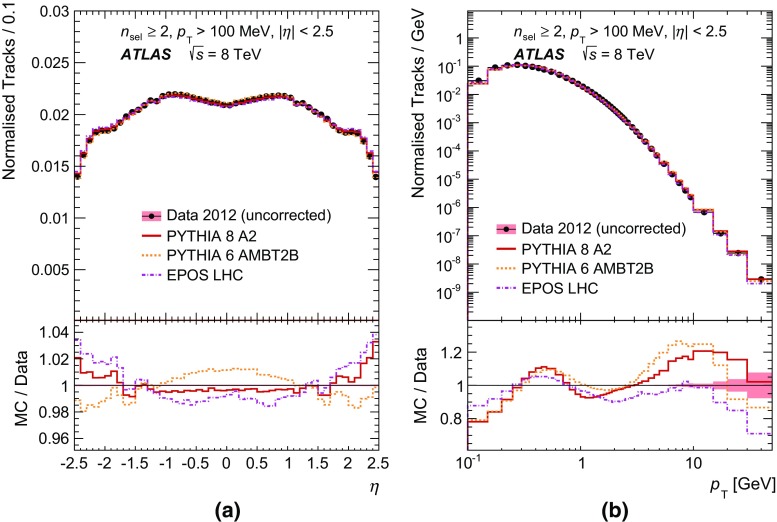
Distribution of the fraction of selected tracks as a function of **a** pseudorapidity, $\eta$, and **b** transverse momentum, $p_{\text {T}}$. The predictions of MC models following detector simulation are compared to the data. Bin entries are scaled by the inverse bin width and the resulting distributions are normalised to unity

To ensure that well-reconstructed tracks were used at this step, the pseudorapidity and transverse momentum must satisfy $|\eta | < 2.5$ and $p_{\text {T}} > 100 \mathrm {\, MeV}$. A number of further quality criteria were also applied. The track must have at least one hit in the pixel detector. A hit in the innermost layer of the pixel detector was required should the extrapolated track have passed through an active region in that layer. At least two, four or six SCT hits are required to be associated with a track for $100 < p_{\text {T}} \le 200 \mathrm {\, MeV}$, $200 < p_{\text {T}} \le 300 \mathrm {\, MeV}$, or $p_{\text {T}} > 300 \mathrm {\, MeV}$, respectively. The SCT hit requirements are relaxed in the event of a track trajectory passing through inactive SCT modules. The probability of the track hypothesis being correct, estimated using the track fit ${\chi }^2$ and $n_{\mathrm {dof}}$, was required to be greater than 0.01 for $p_{\text {T}} > 10 \mathrm {\,GeV}$ in order to remove tracks with a mis-measured high $p_{\text {T}}$ due to interactions with the material or combinatorial fake high-$p_{\text {T}}$ tracks. The distance of closest approach in the transverse ($|d_\mathrm {0}^{\mathrm {PV}} |$) and the longitudinal plane ($|z_\mathrm {0}^{\mathrm {PV}} \cdot \sin {\theta } |$) was also required to be less than 1.5  mm with respect to the primary vertex. These constraints reduce the total fraction of non-primary tracks in the data from around 6 to 2 % (see Sect. 6). The average efficiency to reconstruct a track above $p_{\text {T}} >100 \mathrm {\, MeV}$ is approximately 70 %. The efficiency of the two impact parameter requirements is around 94 %, i.e. applied together they remove approximately 6 % of all tracks that would pass the other track selection criteria.

**Fig. 2:**
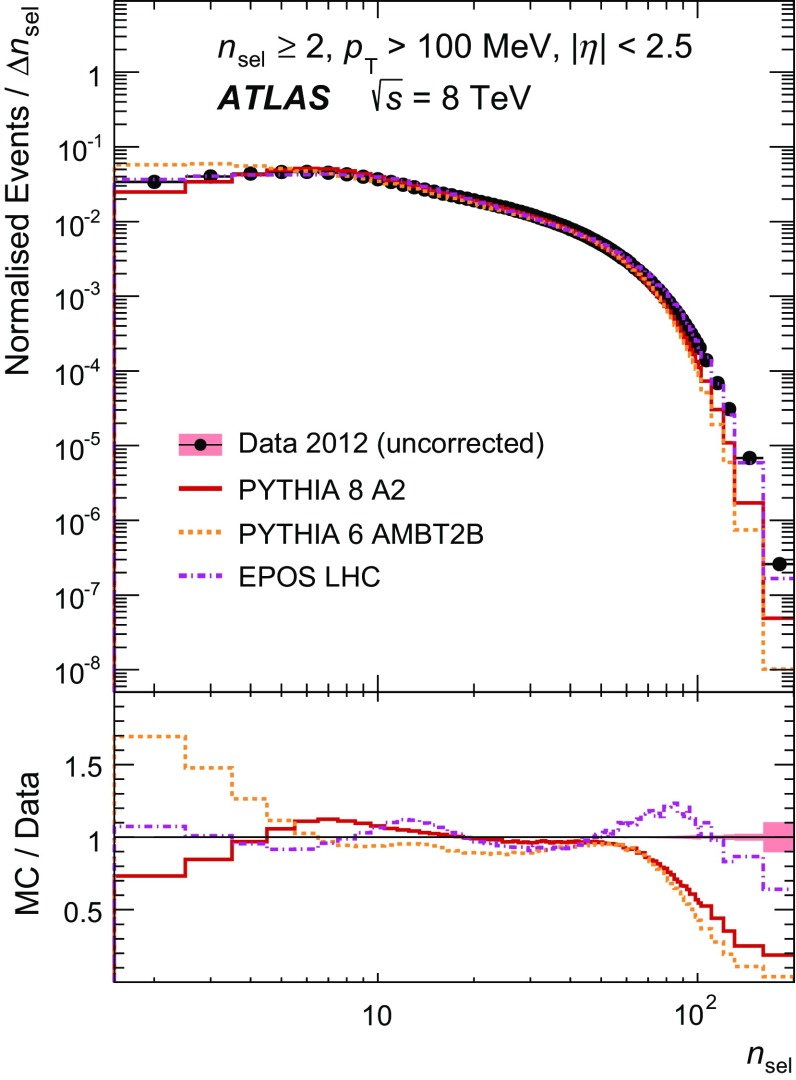
The fraction of selected events as a function of the track multiplicity $n_{\mathrm {sel}}$ per event. The predictions of MC models following detector simulation are compared to the data. Bin entries are scaled by the inverse bin width and the resulting distributions are normalised to unity

Figure 1a shows the normalised distribution of all selected tracks as a function of pseudorapidity in the most inclusive phase space. The models reproduce the data well with discrepancies in $\eta$ at a level of up to 3 %, which stem from the imperfect description of the $p_{\text {T}}$ spectra (Fig. 1b) by the models, where discrepancies of up to $\sim \!30~\%$ are visible. Figure 2 shows the normalised distribution of the fraction of all selected events as a function of track multiplicity per event.

The distributions of the average number of hits per reconstructed track in data and MC simulation as a function of pseudorapidity are shown in Fig. 10 in Appendix A, using events selected for the most inclusive phase space.

## Backgrounds

Background events and tracks can arise from a number of sources, which are described by order of importance.

Corrections were made to the charged-particle spectra to remove the contribution of charged non-primary particles, i.e. those not originating from the *pp* collision. Non-primary particles are mainly due to hadronic interactions, photon conversions and weak decays. MC simulations of the shape of the $d_\mathrm {0}^{\mathrm {PV}}$ distributions were used to quantify the fractions of primaries, non-primaries from electrons and other non-primaries which satisfy the track quality criteria. Using the same method as in Ref. [18], fits were made to the data using $d_\mathrm {0}^{\mathrm {PV}}$ distribution templates, taken from the MC simulation, to assess the fractions of the different classes of charged particles. The fitted impact parameter distributions are shown in Fig. 11 in Appendix B. The total non-primary fraction was about 3 % in the $100< p_{\text {T}} < 150 \mathrm {\, MeV}$ range and about 2 % at higher $p_{\text {T}}$ values. The relative contribution of electrons (including those from rare Dalitz decays) to this fraction was about 35 % at $p_{\text {T}} < 150 \mathrm {\, MeV}$ and dropped below 15 % with rising $p_{\text {T}}$. Systematic uncertainties due to aspects of the template fitting method as well as the choice of MC models were added in quadrature.

In contrast to previous measurements at lower energies [14, 18], and in line with the $13 \mathrm {\, TeV}$ measurement [23], charged particles with a mean lifetime $30< \tau < 300\, \mathrm{ps}$ (mostly charged strange baryons) are considered to be non-stable. The reconstruction efficiency of these short-lived particles and their decay products is strongly momentum-dependent and close to zero for most particles within the measured kinematic range. However, the predicted fraction of the total generated particles associated with charged strange baryons varies with $p_{\text {T}}$ as well as between MC models. For example, the fractions predicted by Pythia 8 A2 and Epos LHC are 5 and 13 %, respectively for $p_{\text {T}} \sim \!5 \mathrm {\,GeV}$. To lower the model dependence on the overall track reconstruction efficiency, the contribution of such particles to the distributions under study was excluded from the measurement definition. The residual small contamination of reconstructed tracks, which is less than 0.01 % in $\eta$ and up to 5 % at high transverse momentum ($30< p_{\text {T}} < 50 \mathrm {\,GeV}$), was estimated from simulation using Epos LHC and subtracted, and a systematic uncertainty was assigned following comparisons of the predictions of different MC models.

Fake tracks are reconstructed either due to detector noise or shared hits from more than one charged particle. These were estimated in simulation to be less than 0.1 % of all tracks.

Beam-induced background, i.e. beam–gas interactions and scattering from up-stream collimators, was estimated using unpaired bunches. Beam-induced backgrounds as well as pile-up contamination were reduced to a negligible level by the track-level and event-level criteria described in Sects. 4 and 5. The cosmic-ray background was found to be negligible using the techniques in Ref. [18].

## Selection efficiencies

In order to obtain inclusive spectra for primary charged particles, the data are corrected from detector level to particle level, using corrections which account for inefficiencies due to trigger selection, vertex and track reconstruction. The methods used to obtain these efficiencies and their systematic uncertainties are described in the following sections.

### Trigger efficiency

The trigger efficiency, $\varepsilon _\mathrm {trig}$, was measured from a data sample selected using a random control trigger in coincidence with colliding bunches with a minimum requirement of two Pixel and three SCT measurements. For this efficiency, the requirement of a reconstructed primary vertex was removed from the selection of events to account for possible correlations between the trigger and vertex reconstruction efficiencies. The trigger efficiency was therefore parameterised as a function of $n_{\mathrm {sel}}^{\mathrm {BS}}$, which is defined as the number of tracks in an event that satisfy all track quality criteria; however, instead of the nominal requirements for the impact parameters $d_\mathrm {0}^{\mathrm {PV}}$ and $z_\mathrm {0}^{\mathrm {PV}}$, only a constraint on the transverse impact parameter with respect to the beam spot, $|d_\mathrm {0}^{\mathrm {BS}} | < 1.8\,\mathrm{mm}$, was applied in order to minimise correlations between the trigger and vertex efficiency corrections.

**Fig. 3:**
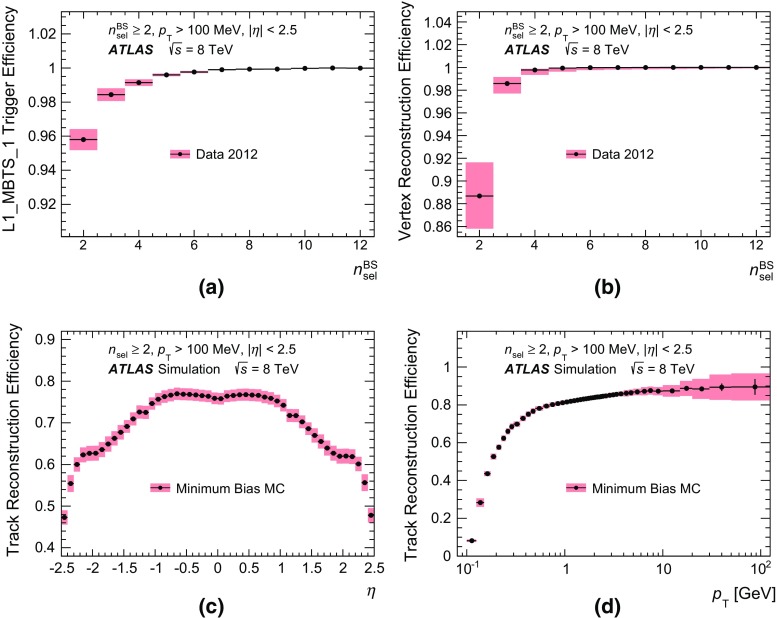
Selection efficiencies for $8 \mathrm {\, TeV}$ data in the most inclusive measured phase space with transverse momentum $p_{\text {T}} > 100 \mathrm {\, MeV}$: **a** The L1_MBTS_1 trigger efficiency as a function of the number of selected tracks, $n_{\mathrm {sel}}^{\mathrm {BS}}$. L1_MBTS_1 is the requirement that in at least one module of the minimum-bias trigger scintillators a signal above threshold was registered. **b** The vertex reconstruction efficiency as a function of the number of selected tracks, $n_{\mathrm {sel}}^{\mathrm {BS}}$. **c** The track reconstruction efficiency as a function of the pseudorapidity, $\eta$. **d** The track reconstruction efficiency as a function of the transverse momentum, $p_{\text {T}}$. The *shaded areas* represent the sum of systematic and statistical errors

The trigger efficiency was calculated as the ratio of events triggered by the control trigger, in which the MBTS trigger also accepted the event, to the total number of triggered events in the control sample. It was determined separately for the trigger requirement in which the signal in at least one or two of the MBTS modules was above threshold. In order to maximise the recorded data yield, both triggers were combined such that either of the two triggers was required to trigger the event, in which case the corresponding trigger efficiency was applied; this was done due to pre-scale factors that evolved differently for each trigger during the run. The result for the trigger requirement in which the signal in at least one of the MBTS modules was above threshold is presented in Fig. 3a as a function of $n_{\mathrm {sel}}^{\mathrm {BS}}$ for the most inclusive phase space. In the $p_{\text {T}} > 500 \mathrm {\, MeV}$ phase space, the efficiency was measured to be above 98 % for $n_{\mathrm {sel}}^{\mathrm {BS}} = 1$ and it rises more rapidly to 100 % at higher track multiplicities than in the most inclusive phase space. The efficiency for the trigger requirement in which a signal above threshold was required in at least two of the MBTS modules is lower by about 4 % for the ($n_{\mathrm {sel}}^{\mathrm {BS}} = 2, \ p_{\text {T}} > 100 \mathrm {\, MeV}$) event category, and about 2 % lower for events with $n_{\mathrm {sel}}^{\mathrm {BS}} = 1$ and $p_{\text {T}} > 500 \mathrm {\, MeV}$. It rises more slowly to 100 % as a function of $n_{\mathrm {sel}}^{\mathrm {BS}}$ for both $p_{\text {T}}$ requirements. These additional results are shown in Fig. 12 in Appendix C.

The trigger requirement was found to introduce no observable bias in the $p_{\text {T}}$ and $\eta$ distributions of selected tracks beyond the statistical uncertainties of the data recorded with the control trigger. The systematic uncertainties shown in Fig. 3a due to beam-induced background and tracks from secondary particles were estimated from differences in the trigger efficiency by varying the impact parameter requirements in the track selection. The total systematic uncertainty on the trigger efficiency in the $n_{\mathrm {sel}}^{\mathrm {BS}} \ge 2, \ p_{\text {T}} \ge 100 \mathrm {\, MeV}$ phase space was 0.7 % for $n_{\mathrm {sel}}^{\mathrm {BS}} = 2$, decreasing rapidly at higher track multiplicities. This uncertainty is very small compared to those from other sources.

### Vertex reconstruction efficiency

The vertex reconstruction efficiency, $\varepsilon _\mathrm {vtx}$, was determined for data and MC simulation from the ratio of selected events which satisfy the trigger requirement and contain a reconstructed vertex to the total number of triggered events. The expected contribution from beam-induced background events is estimated using the same method as described in Ref. [18] and subtracted before measuring the efficiency. The vertex reconstruction efficiency was parameterised as a function of $n_{\mathrm {sel}}^{\mathrm {BS}}$, using the same track quality criteria with modified impact parameter constraints as for the trigger efficiency.

The result is shown in Fig. 3b as a function of $n_{\mathrm {sel}}^{\mathrm {BS}}$ for events in the most inclusive phase space with $p_{\text {T}} > 100 \mathrm {\, MeV}$. The efficiency was measured to be approximately 89 % for $n_{\mathrm {sel}}^{\mathrm {BS}} = 2$, rapidly rising to 100 % at higher track multiplicities. For the $p_{\text {T}} > 500 \mathrm {\, MeV}$ phase space, the result is given in Fig. 13a in Appendix D. For events with $n_{\mathrm {sel}}^{\mathrm {BS}} = 2$ in the $p_{\text {T}} > 100 \mathrm {\, MeV}$ phase space, the efficiency was parameterised as a function of the minimum difference in longitudinal impact parameter ($\Delta z^\mathrm {min}_0$) of track pairs, as well as the minimum transverse momentum ($p_{\text {T}}^{\mathrm {min}}$) of selected tracks in the event. For events with $n_{\mathrm {sel}}^{\mathrm {BS}} = 1$ in the $p_{\text {T}} > 500 \mathrm {\, MeV}$ phase space, the efficiency was parameterised as a function of $\eta$ of the single track.

The systematic uncertainty was estimated from adding in quadrature the difference between the nominal vertex reconstruction efficiency, measured with beam-induced background removal, and either (1) the vertex reconstruction efficiency measured without beam-induced background removal, or (2) the vertex reconstruction efficiency with a modified impact parameter constraint. The total uncertainty is below 3 % for $n_{\mathrm {sel}}^{\mathrm {BS}} = 2$ in the most inclusive phase space, rapidly decreasing at higher track multiplicities. This uncertainty is small compared to those from other sources, except at very low track multiplicities.

### Track reconstruction efficiency

The primary track reconstruction efficiency, $\varepsilon _\mathrm {trk}$, was determined from MC simulation and parameterised in two-dimensional bins of $p_{\text {T}}$ and $\eta$. This efficiency includes the efficiency of the track selection requirements (see Sect. 5). It is defined as the ratio:

1 $$\begin{aligned} \varepsilon _\mathrm {trk} (p_{\text {T}},\eta ) \ = \ \frac{N_{\mathrm {rec}}^{\mathrm {matched}} (p_{\text {T}}^{\mathrm {gen}},\eta ^{\mathrm {gen}})}{N_{\mathrm {gen}} (p_{\text {T}}^{\mathrm {gen}},\eta ^{\mathrm {gen}})} , \end{aligned}$$

where $p_{\text {T}}^{\mathrm {gen}}$ and $\eta ^{\mathrm {gen}}$ are properties of the generated particle, $N_{\mathrm {rec}}^{\mathrm {matched}} (p_{\text {T}}^{\mathrm {gen}},\eta ^{\mathrm {gen}})$ is the number of reconstructed tracks matched to a generated primary charged particle in a $(p_{\text {T}}^{\mathrm {gen}},\eta ^{\mathrm {gen}})$ bin, and $N_{\mathrm {gen}} (p_{\text {T}}^{\mathrm {gen}},\eta ^{\mathrm {gen}})$ is the number of generated primary charged particles in that bin. A track is considered matched to a generated particle if that particle has the smallest angular distance $\Delta R$ to the track, if $\Delta R < 0.15$, and if the particle trajectory is compatible with the position of at least one pixel hit of the associated track.

The resulting reconstruction efficiency as a function of $\eta$ integrated over $p_{\text {T}}$ is shown in Fig. 3c for the most inclusive phase space, and in Fig. 13b in Appendix D for the phase space given by $n_{\mathrm {sel}} \ge 1$ and $p_{\text {T}} > 500 \mathrm {\, MeV}$. The shape of the $\eta$ distribution is strongly affected by the amount of material traversed by charged particles, in particular the passive material in supporting structures between the Pixel and SCT detector. A larger amount of passive material is located at high $|\eta |$ and increases the probability of particles to undergo particle–matter interactions such as hadronic interactions, which reduces the track reconstruction efficiency. The approximately constant efficiency at $|\eta | \sim \!2.1$ is due to the particles passing through an increasing number of layers in the ID end-cap. Figure 3d shows the efficiency as a function of $p_{\text {T}}$ integrated over $\eta$. The $p_{\text {T}}$ dependence is largely due to the requirement on the minimum number of silicon hits in the track reconstruction algorithms, which is less likely to be fulfilled by lower-$p_{\text {T}}$ tracks.

As the track reconstruction efficiency is determined from MC simulation, its systematic uncertainties result from model dependencies and from the uncertainty of the detector material description used in the simulation. Since the generated particle composition and the reconstructed track composition differs between MC tunes, a small model-dependence of the track reconstruction efficiency can be observed, leading to an additional systematic uncertainty due to the particle composition. The impact of the choice of physics models for hadronic interactions in Geant 4 simulation is also taken into account.

The amount of material within the ID was constrained to within $\pm 5~\%$, based on extensive studies of material interactions [39]. The systematic uncertainties on the track reconstruction efficiencies were obtained by comparing the predictions of simulations which assume the nominal ID material distribution with two special simulations in which the assumed material was varied. For one simulation, the amount of non-sensitive ID material was increased by 5 % in terms of radiation length $X_0$. In the other, the Pixel service material was increased by 10 % in $X_0$. These studies give rise to an average systematic uncertainty on the track reconstruction efficiency of 1.6–1.7 % in the central region and up to 3.5 % in the forward region, with larger uncertainties up to 8 % for particles with very low transverse momenta of $p_{\text {T}} < 150 \mathrm {\, MeV}$. This is the dominant contribution to the total systematic uncertainty in most regions of the measured distributions. The reduction of this uncertainty with respect to measurements at $\sqrt{s} = 7 \mathrm {\, TeV}$, due to our improved knowledge of the ID material distribution, is about 50 % in the central region and rises to as much as 65 % in the forward region.

Systematic uncertainties due to simulation of the efficiency of the requirements on the number of hits associated with a track, the impact parameter requirements, and the efficiency of the track-fit $\chi ^2$ probability requirement were found by comparing each selection efficiency in data and MC simulation. The sum in quadrature of these uncertainties varies between 0.5 and 1.6 % for all $\eta$ values and $p_{\text {T}} < 10 \mathrm {\,GeV}$, and increases to as much as 8 % for high-momentum tracks above $p_{\text {T}} > 30 \mathrm {\,GeV}$ in the most forward regions.

The systematic uncertainty due to different fractions of positively and negatively charged tracks in data and MC simulation was found to be negligible.

The total uncertainty of the track reconstruction efficiency, shown in Fig. 3c, d, was obtained by adding all effects in quadrature and is dominated by the uncertainty from the material description.

## Correction procedure

In order to obtain inclusive particle-level distributions, all measured detector-level distributions were corrected by an event-by-event weight, and track distributions were additionally corrected by a track-by-track weight, to compensate for the inefficiencies of the data selection and the reconstruction algorithms, as well as for contaminations due to various sources of background. Furthermore, a Bayesian unfolding procedure [40] was applied to compensate for migration and resolution effects in the observed multiplicity and transverse momentum distributions.

### Event and track weights

All selected events were corrected with an event-by-event weight to compensate for the inefficiencies of the MBTS trigger selection and the vertex reconstruction algorithm. The total event weight $w_\mathrm {ev}$ is parameterised as:

2 $$\begin{aligned} w_\mathrm {ev}(n_{\mathrm {sel}}^{\mathrm {BS}},x) = \frac{1}{{\varepsilon _\mathrm {trig} (n_{\mathrm {sel}}^{\mathrm {BS}})} \cdot {\varepsilon _\mathrm {vtx} (n_{\mathrm {sel}}^{\mathrm {BS}},x)}} . \end{aligned}$$

The parameter *x* represents a combination of $p_{\text {T}}^{\mathrm {min}}$ for all selected tracks, the minimum difference in longitudinal impact parameter ($\Delta z^\mathrm {min}_0$) for track pairs, and $\eta$ of a single track (for events with only one selected track), as described in Sect. 7.2. In addition, the MC simulation events were weighted such that the vertex *z*-distribution agrees with that observed in data.

Furthermore, a track-by-track weight, $w_\mathrm {trk}(p_{\text {T}},\eta )$, was estimated for each selected track as a function of the transverse momentum and pseudorapidity assigned to the track, based on the track reconstruction efficiency, $\varepsilon _\mathrm {trk} (p_{\text {T}},\eta )$, the fraction of non-primary tracks, $f_\mathrm {nonp} (p_{\text {T}},\eta )$, the fraction of tracks associated with a strange baryon, $f_\mathrm {sb} (p_{\text {T}},\eta )$, and the fraction of additional tracks corresponding to particles outside the kinematic range but migrating into the kinematic region due to resolution effects, $f_\mathrm {okr} (p_{\text {T}},\eta )$:

3 $$\begin{aligned} w_\mathrm {trk}(p_{\text {T}},\eta ) = \frac{1 - f_\mathrm {nonp} (p_{\text {T}},\eta ) - f_\mathrm {sb} (p_{\text {T}},\eta ) - f_\mathrm {okr} (p_{\text {T}},\eta )}{\varepsilon _\mathrm {trk} (p_{\text {T}},\eta )} . \end{aligned}$$

The quantities $\varepsilon _\mathrm {trk} (p_{\text {T}},\eta )$, $f_\mathrm {sb} (p_{\text {T}},\eta )$ and $f_\mathrm {okr} (p_{\text {T}},\eta )$ were evaluated using MC models. The quantification of the contamination due to non-primary tracks and strange baryons is described in Sect. 6.

### Correction to ${\mathrm {d} N_{\mathrm {ev}}}/{\mathrm {d} n_{\mathrm {ch}}}$

Only the event-level corrections for the trigger and vertex inefficiencies were applied to the charged-particle multiplicity distribution. Thereafter, a Bayesian unfolding process was applied to correct the observed multiplicity $n_{\mathrm {sel}}$ to the true number of primary charged particles $n_{\mathrm {ch}}$. This is the same procedure as was applied in Ref. [18], using five iterations in the $p_{\text {T}} > 100 \mathrm {\, MeV}$ phase space, and four iterations for $p_{\text {T}} > 500 \mathrm {\, MeV}$. After the unfolding, a correction was made to the resulting primary-charged-particle multiplicity distribution to account for events migrating out of the multiplicity range required by the phase space.

The corrected distribution ${\mathrm {d} N_{\mathrm {ev}}}/{\mathrm {d} n_{\mathrm {ch}}}$ was integrated over $n_{\mathrm {ch}}$ to give the total number of events $N_{\mathrm {ev}}$. The quantity $N_{\mathrm {ev}}$ was then used to normalise the distributions ${1}/({2 \pi p_{\text {T}} {N_{\mathrm {ev}}}}) \cdot {\mathrm {d}^2 N_{\mathrm {ch}}}/({\mathrm {d} \eta \, \mathrm {d} p_{\text {T}}})$ and ${1}/{N_{\mathrm {ev}}} \cdot {\mathrm {d} N_{\mathrm {ch}}}/{\mathrm {d} \eta }$, as well as the multiplicity distribution itself, ${1}/{N_{\mathrm {ev}}} \cdot {{\mathrm {d} N_{\mathrm {ev}}}/{\mathrm {d} n_{\mathrm {ch}}}}$.

### Correction to ${1}/({2 \pi p_{\text {T}} {N_{\mathrm {ev}}}}) \cdot {\mathrm {d}^2 N_{\mathrm {ch}}}/({\mathrm {d} \eta \, \mathrm {d} p_{\text {T}}})$ and ${1}/{N_{\mathrm {ev}}} \cdot {\mathrm {d} N_{\mathrm {ch}}}/{\mathrm {d} \eta }$

Corrections were made for trigger requirements, vertex and track reconstruction inefficiencies, migration effects due to the resolution of reconstructed track parameters, and the influence of non-primary tracks. A Bayesian unfolding method, similar to that used to correct the $n_{\mathrm {ch}}$ spectra, was then employed to give the ${1}/({2 \pi p_{\text {T}} {N_{\mathrm {ev}}}}) \cdot {\mathrm {d}^2 N_{\mathrm {ch}}}/({\mathrm {d} \eta \, \mathrm {d} p_{\text {T}}})$ distribution, using four iterations in the $p_{\text {T}} > 100 \mathrm {\, MeV}$ phase space, and up to five iterations for $p_{\text {T}} > 500 \mathrm {\, MeV}$. Fake high-$p_{\text {T}}$ tracks are already suppressed by the $\chi ^2$ probability requirement in the track selection, and remaining fake tracks are also unfolded for by this procedure.

### Correction to $\langle p_{\text {T}} \rangle$ versus $n_{\mathrm {ch}}$

The $\langle p_{\text {T}} \rangle$ versus $n_{\mathrm {ch}}$ distribution was evaluated in the following way. Corrections were made to two separate spectra: the distribution of the $\Sigma _{i} p_{\text {T}} (i)$ (where the summation is made over the transverse momentum of all selected tracks in all events within a certain range of track multiplicity) versus the number of selected tracks per event, and the distribution of the sum of all selected tracks in all events within a certain range of track multiplicity versus the number of selected tracks per event. The distributions were first corrected with the appropriate track weights, which was followed by Bayesian unfolding. Finally, the ratio of the two spectra was taken to obtain the corrected $\langle p_{\text {T}} \rangle$ versus $n_{\mathrm {ch}}$ distribution.

## Systematic uncertainties

In the analysis procedure, most of the individual sources of systematic uncertainty given below were applied separately as variations of the event or track weights, producing new distributions which were used to obtain alternative versions of the final corrected and unfolded results. Other sources were assessed by varying the input distributions (e.g. in $n_{\mathrm {ch}}$ distributions, the multiplicity of each event was randomly varied with probabilities corresponding to the uncertainties on the track reconstruction efficiencies) or unfolding matrices (using statistical variations, or matrices obtained from different MC generators) which were used for the Bayesian unfolding procedure, thus producing the alternative results. In all these cases, the differences from the nominal distributions were taken as systematic uncertainties.

The following sources of systematic uncertainty in the corrected distributions were considered.

- Incomplete knowledge of the material distribution in the ID affects the measured spectra by between 1 and 8 %. This source of systematic uncertainty is described in detail in Sect. 7.3. The total uncertainty due to the material distribution is typically less than 5 % over all distributions other than at $p_{\text {T}} < 150 \mathrm {\, MeV}$ in the transverse momentum spectrum, at $n_{\mathrm {ch}} \ge 120$ in the multiplicity spectrum of the $p_{\text {T}} > 100 \mathrm {\, MeV}$ phase space, and at $n_{\mathrm {ch}} \ge 70$ in the multiplicity spectrum of the $p_{\text {T}} > 500 \mathrm {\, MeV}$ phase space. This is the dominant uncertainty on ${1}/{N_{\mathrm {ev}}} \cdot {\mathrm {d} N_{\mathrm {ch}}}/{\mathrm {d} \eta }$, and the leading or next-to-leading uncertainty in all other distributions.
- Different $p_{\text {T}}$ spectra in the MC models and data lead to differences of up to 2 % in the average track reconstruction efficiency per $n_{\mathrm {ch}}$ interval. For the final ${\mathrm {d} N_{\mathrm {ev}}}/{\mathrm {d} n_{\mathrm {ch}}}$ spectra in the most inclusive phase space, this effect becomes as large as 12 % at the highest multiplicities.
- The relative uncertainty on the fraction of non-primary tracks is 15 %, while the relative uncertainty on the fraction of reconstructed strange baryons is 50 %, as described in Sect. 6. The total uncertainty of both sources in the corrected distributions is 3.5 % or smaller and is not a dominant uncertainty in any region.
- Different particle types have different reconstruction efficiencies. For example, at $p_{\text {T}} \sim \!1 \mathrm {\,GeV}$ the reconstruction efficiency of charged pions is $\sim \!82~\%$, whereas for kaons and protons it is $\sim$80 and $\sim$75 %, respectively. Although the MC generators give consistent efficiencies, the relative fractions of these generated particles vary between the models. For example, Pythia 8 A2 (Epos LHC) gives fractions of 77 % (72 %), 14 % (18 %) and 9 % (10 %) for generated pions, kaons and protons, respectively, at $p_{\text {T}} \sim \!1 \mathrm {\,GeV}$. Differences in particle composition therefore lead to an uncertainty on the overall track reconstruction efficiency, which varies between 0.2 and 1 % for the corrected distributions. This is not a dominant uncertainty in any region.
- Systematic uncertainties on the overall track reconstruction efficiency that are associated with the choice of track–particle matching algorithms (0.4 %) and the choice of physics models for MC simulation (0.3 %) are also accounted for, and are not a dominant uncertainty in any region of the corrected distributions.
- To account for momentum resolution differences between data and MC simulation, which can arise, for example, via imperfect knowledge of the detector alignment, an uncertainty of 5 % was assigned to tracks with $p_{\text {T}} < 150 \mathrm {\, MeV}$. At higher values of $p_{\text {T}}$ a one-sided uncertainty of −7 % for $10< p_{\text {T}} < 30 \mathrm {\,GeV}$ and −9 % for $p_{\text {T}} > 30 \mathrm {\,GeV}$ tracks was assigned, as in the previous work at $\sqrt{s} = 7 \mathrm {\, TeV}$ [18], due to the steeply falling $p_{\text {T}}$ spectrum in combination with the lower momentum resolution in data. This is combined with a one-sided uncertainty due to the estimated fraction of mis-measured high-$p_{\text {T}}$ tracks, which increases with transverse momentum to as much as −16 % for $p_{\text {T}} > 30 \mathrm {\,GeV}$ tracks. The effect on the corrected distributions is typically negligible, except in the corrected $p_{\text {T}}$ spectra.
- Differences in the efficiencies of track quality criteria between data and MC simulation give rise to systematic uncertainties in the final spectra which are typically below 1 %, except at transverse momenta above $10 \mathrm {\,GeV}$ and at high multiplicities, reaching as much as 6 and 5 %, respectively. However, this remains a small uncertainty compared to those from other sources in the same regions.
- Event-level uncertainties on the trigger efficiency and vertex reconstruction efficiency give rise to systematic uncertainties of up to 3 % in the lowest multiplicity intervals of the ${\mathrm {d} N_{\mathrm {ev}}}/{\mathrm {d} n_{\mathrm {ch}}}$ spectra. However, even in these regions this uncertainty is dominated by other sources.
- For each presented distribution, closure tests were performed. A closure test applies the full nominal correction procedure to reconstructed MC simulation events and quantifies the degree to which the generated particle-level distribution is reproduced. The degree of non-closure is typically less than 1 % and/or below the level of statistical uncertainties. Larger non-closures were found for the lower end of the $p_{\text {T}}$ spectrum, $100< p_{\text {T}} < 150 \mathrm {\, MeV}$, where the non-closure is found to be 6 % due to momentum resolution effects, and in the low-multiplicity region of the average transverse momentum $\langle p_{\text {T}} \rangle$ as a function of $n_{\mathrm {ch}}$, with up to 4 % non-closure in the $p_{\text {T}} > 100 \mathrm {\, MeV}$ phase space due to assumptions made in the unfolding procedure. All of these non-closures were taken into account as an additional source of systematic uncertainty.
- Uncertainties associated with the unfolding technique are estimated as the degree of non-closure following a modified correction procedure, i.e. obtained in corrected multiplicities after varying the input spectra and unfolding matrix. This is the dominant uncertainty on ${1}/({2 \pi p_{\text {T}} {N_{\mathrm {ev}}}}) \cdot {\mathrm {d}^2 N_{\mathrm {ch}}}/({\mathrm {d} \eta \, \mathrm {d} p_{\text {T}}})$ for transverse momentum values of $p_{\text {T}} > 10 \mathrm {\,GeV}$, for which the uncertainty varies from 6 to 20 %, as well as over the entire range of $\langle p_{\text {T}} \rangle$ versus $n_{\mathrm {ch}}$. It is also the largest uncertainty in the low and high multiplicity regions of ${\mathrm {d} N_{\mathrm {ev}}}/{\mathrm {d} n_{\mathrm {ch}}}$, for which it has values between 1 and 12 %.

All sources of systematic uncertainty are added in quadrature, thus yielding the total systematic uncertainties which are shown as shaded areas in the figures in the next section. The total systematic uncertainties in the two most inclusive phase spaces, at $p_{\text {T}} >100 \mathrm {\, MeV}$ ($p_{\text {T}} > 500 \mathrm {\, MeV})$, range from 1.8 to 3.6 % (1.3 to 2.1 %) in the final ${1}/{N_{\mathrm {ev}}} \cdot {\mathrm {d} N_{\mathrm {ch}}}/{\mathrm {d} \eta }$ distributions, from 1.6 to 30 % in the final ${1}/({2 \pi p_{\text {T}} {N_{\mathrm {ev}}}}) \cdot {\mathrm {d}^2 N_{\mathrm {ch}}}/({\mathrm {d} \eta \, \mathrm {d} p_{\text {T}}})$ distributions, from 3 to 21 % (2 to 16 %) in the final ${\mathrm {d} N_{\mathrm {ev}}}/{\mathrm {d} n_{\mathrm {ch}}}$ spectra, and from 1.3 to 4 % (0.5 to 2.2 %) in the final $\langle p_{\text {T}} \rangle$ versus $n_{\mathrm {ch}}$ distributions. The lowest uncertainties within these ranges are found at central pseudorapidity ($\eta =0$), around medium transverse momentum values ($p_{\text {T}} \sim \!1 \mathrm {\,GeV}$), and around average multiplicity values of $n_{\mathrm {ch}} \sim \!20$.

**Fig. 4:**
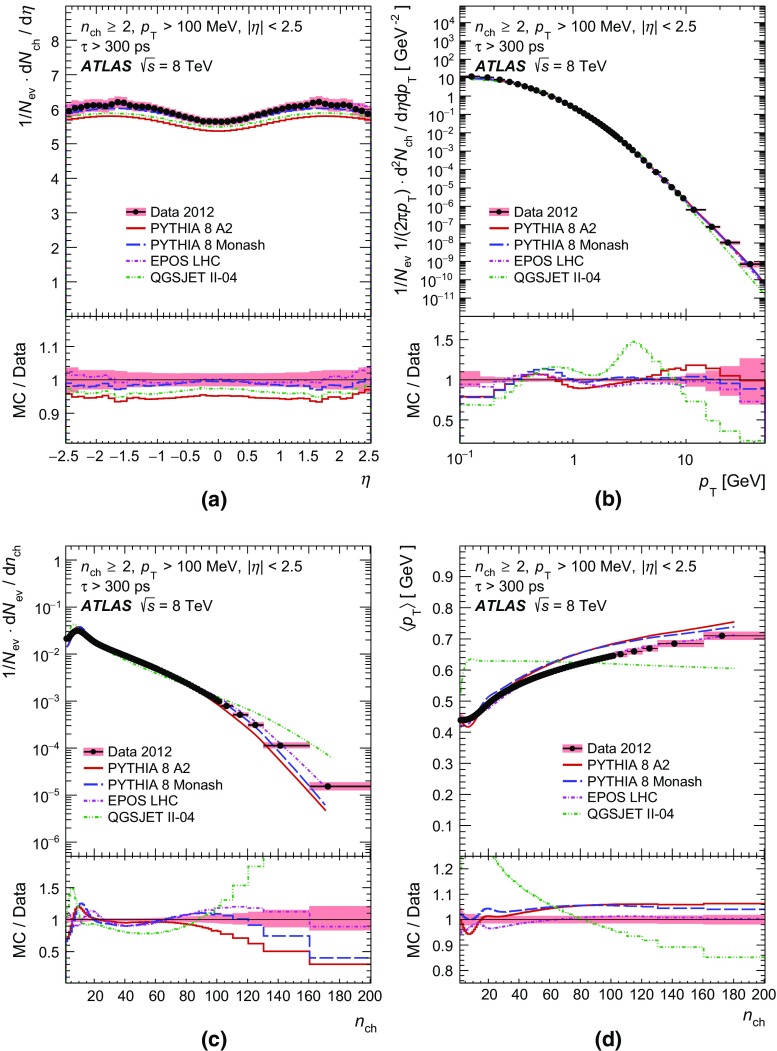
Distributions of primary charged particles in events for which $n_{\mathrm {ch}} \ge 2$, $p_{\text {T}} > 100 \mathrm {\, MeV}$ and $|\eta | < 2.5$ as a function of **a** pseudorapidity, $\eta$, **b** transverse momentum, $p_{\text {T}}$, **c** multiplicity, $n_{\mathrm {ch}}$, and **d** average transverse momentum, $\langle p_{\text {T}} \rangle$, versus multiplicity. The data, represented by *dots*, are compared to various particle-level MC predictions, which are shown by *curves*. The *shaded areas* around the data points represent the total statistical and systematic uncertainties added in quadrature

**Fig. 5:**
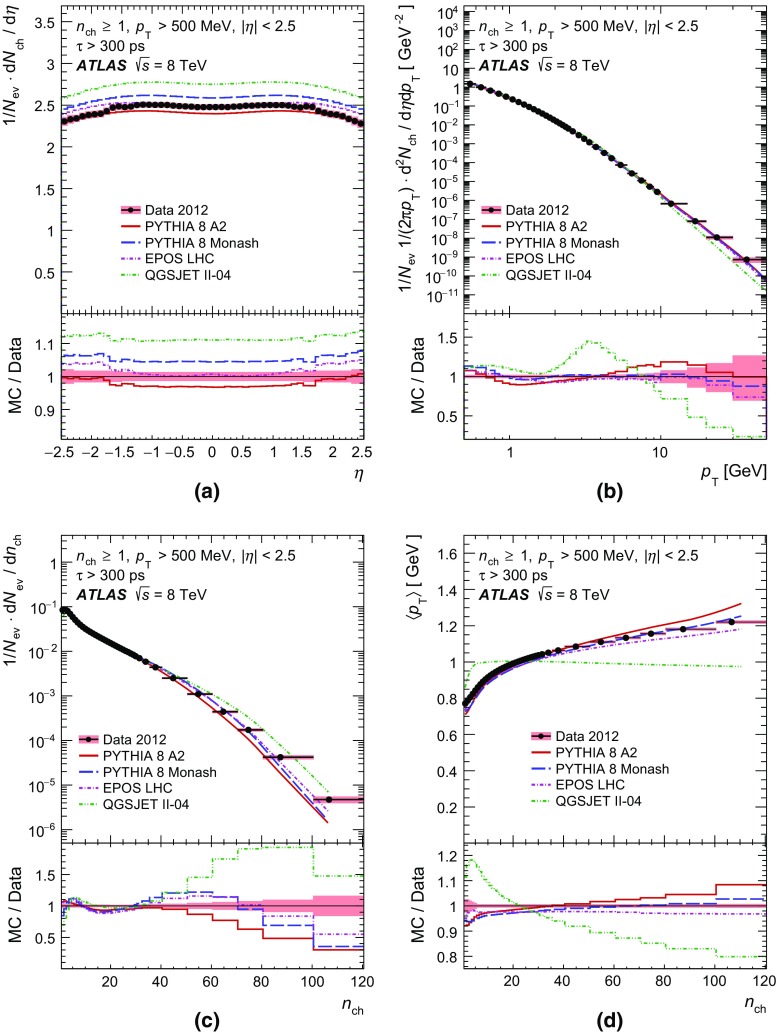
Distributions of primary charged particles in events for which $n_{\mathrm {ch}} \ge 1$, $p_{\text {T}} > 500 \mathrm {\, MeV}$ and $|\eta | < 2.5$ as a function of **a** pseudorapidity, $\eta$, **b** transverse momentum, $p_{\text {T}}$, **c** multiplicity, $n_{\mathrm {ch}}$, and **d** average transverse momentum, $\langle p_{\text {T}} \rangle$, versus multiplicity. The data, represented by *dots*, are compared to various particle-level MC predictions, which are shown by *curves*. The *shaded areas* around the data points represent the total statistical and systematic uncertainties added in quadrature

**Fig. 6:**
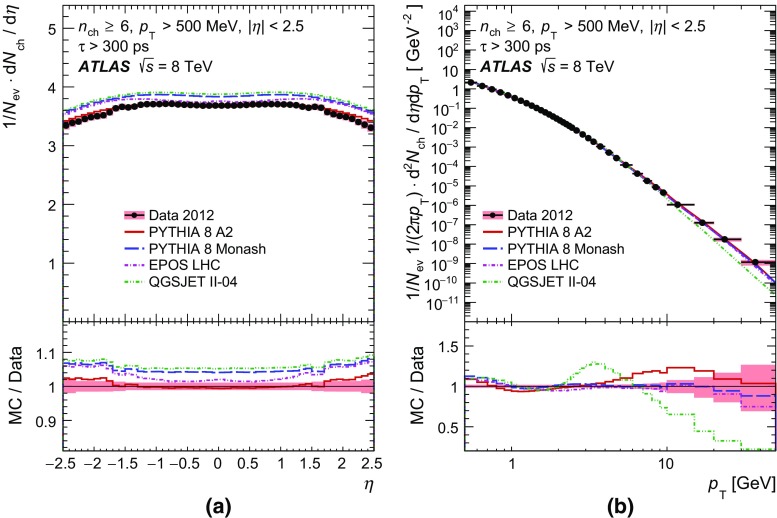
Distributions of primary charged particles in events for which $n_{\mathrm {ch}} \ge 6$, $p_{\text {T}} > 500 \mathrm {\, MeV}$ and $|\eta | < 2.5$ as a function of **a** pseudorapidity, $\eta$, and **b** transverse momentum, $p_{\text {T}}$. The data, represented by *dots*, are compared to various particle-level MC predictions, which are shown by *curves*. The *shaded areas* around the data points represent the total statistical and systematic uncertainties added in quadrature

**Fig. 7:**
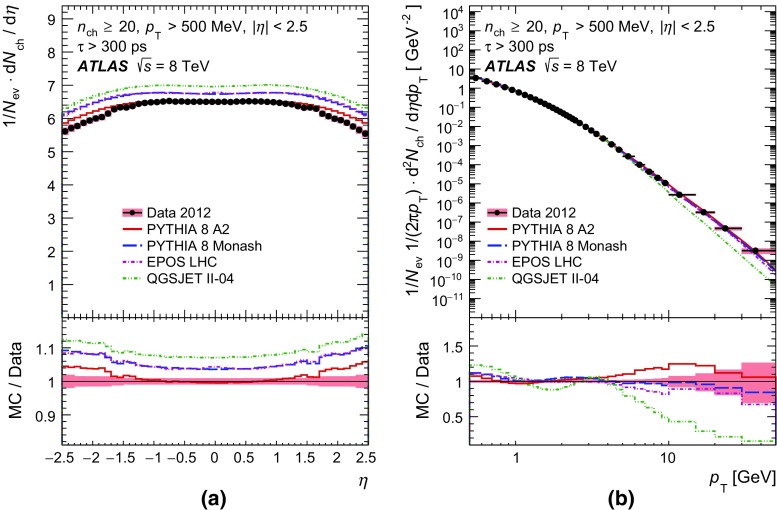
Distributions of primary charged particles in events for which $n_{\mathrm {ch}} \ge 20$, $p_{\text {T}} > 500 \mathrm {\, MeV}$ and $|\eta | < 2.5$ as a function of **a** pseudorapidity, $\eta$, and **b** transverse momentum, $p_{\text {T}}$. The data, represented by *dots*, are compared to various particle-level MC predictions, which are shown by *curves*. The *shaded areas* around the data points represent the total statistical and systematic uncertainties added in quadrature

**Fig. 8:**
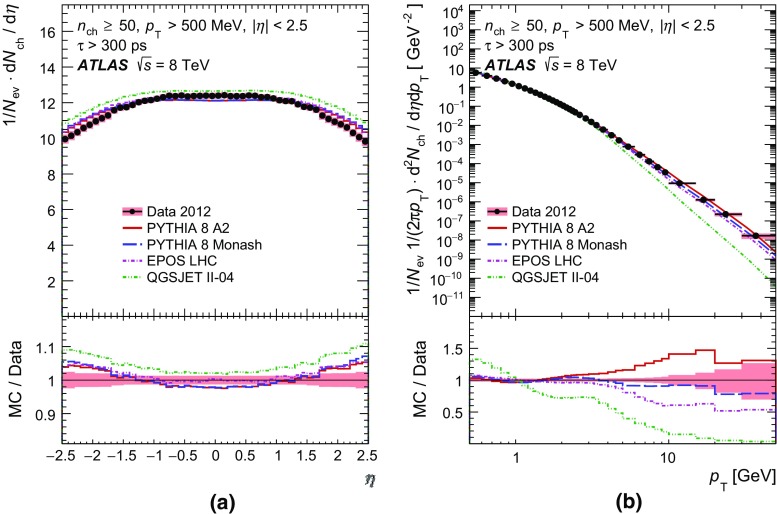
Distributions of primary charged particles in events for which $n_{\mathrm {ch}} \ge 50$, $p_{\text {T}} > 500 \mathrm {\, MeV}$ and $|\eta | < 2.5$ as a function of **a** pseudorapidity, $\eta$, and **b** transverse momentum, $p_{\text {T}}$. The data, represented by *dots*, are compared to various particle-level MC predictions, which are shown by *curves*. The *shaded areas* around the data points represent the total statistical and systematic uncertainties added in quadrature

## Results

Distributions of primary-charged-particle pseudorapidity, ${1}/{N_{\mathrm {ev}}} \cdot {\mathrm {d} N_{\mathrm {ch}}}/{\mathrm {d} \eta }$, are given in Figs. 4a, 5a, 6a, 7a and 8a for all measured phase spaces. The distribution corresponding to the phase space $n_{\mathrm {ch}} \ge 2$ and $p_{\text {T}} > 100 \mathrm {\, MeV}$ rises as $|\eta |$ increases, peaking at $|\eta | \sim \!2$ before falling. For the phase space $n_{\mathrm {ch}} \ge 1$ and $p_{\text {T}} > 500 \mathrm {\, MeV}$, the distribution is approximately constant for $|\eta |<2$ and falls at higher $|\eta |$. A similar shape is seen for the phase spaces requiring a higher multiplicity ($n_{\mathrm {ch}} \ge 6,20,50$) with the extent of the plateau becoming shorter as the multiplicity threshold is raised. A small apparent structure in the distributions of the central values of the data points occurs at values of $|\eta | \sim \!1.7$. This is due to systematic effects in the track reconstruction efficiency which arises due to assumptions on the ID material composition, and is thus covered by the total systematic uncertainty (see Sect. 9).

The distribution corresponding to the phase space $n_{\mathrm {ch}} \ge 2$ and $p_{\text {T}} > 100 \mathrm {\, MeV}$ is well described by Epos LHC and Pythia 8 Monash but is underestimated by Pythia 8 A2 and Qgsjet-II.^5^ For the phase space $n_{\mathrm {ch}} \ge 1$ and $p_{\text {T}} > 500 \mathrm {\, MeV}$, Epos LHC overestimates the distribution at values of $|\eta | > 1.7$ and describes the data well for the rest of the pseudorapidity range. The data are overestimated by the Qgsjet-II and Pythia 8 Monash calculations and underestimated by the Pythia 8 A2 prediction. All models overestimate the overall yield for the phase spaces $n_{\mathrm {ch}} \ge 6,20$ although Pythia 8 A2 describes the plateau in the central region well. For the largest multiplicity threshold ($n_{\mathrm {ch}} \ge 50$) all of the models overestimate the data at $|\eta | > 1.7$ but provide a better description in the central region.

Figures 4b, 5b, 6b, 7b and 8b show distributions of primary-charged-particle transverse momentum, ${1}/({2 \pi p_{\text {T}} {N_{\mathrm {ev}}}}) \cdot {\mathrm {d}^2 N_{\mathrm {ch}}}/({\mathrm {d} \eta \, \mathrm {d} p_{\text {T}}})$, for various phase spaces. No model is fully consistent with the distributions, although above $1 \mathrm {\,GeV}$ the Pythia 8 Monash predictions agree well with the data. This is also the only model which gives a fair description of the data corresponding to the highest multiplicity threshold with $n_{\mathrm {ch}} \ge 50$ and $p_{\text {T}} > 500 \mathrm {\, MeV}$, where all other models show large deviations as $p_{\text {T}}$ increases. The Epos LHC predictions give the best description of the data corresponding to the phase space $n_{\mathrm {ch}} \ge 2$ and $p_{\text {T}} > 100 \mathrm {\, MeV}$, particularly at transverse momenta below $1 \mathrm {\,GeV}$, while the other models underestimate the data at the lowest $p_{\text {T}}$ values. Epos LHC provides fair predictions for the phase spaces $n_{\mathrm {ch}} \ge 1, 6$ and $p_{\text {T}} > 500 \mathrm {\, MeV}$, but for the higher multiplicity thresholds ($n_{\mathrm {ch}} \ge 20$ and 50) deviations from the data are seen at high transverse momenta. Pythia 8 A2 gives fair descriptions of the data below $6 \mathrm {\,GeV}$, yet shows deviations of up to 30 % around $p_{\text {T}} \sim \!10 \mathrm {\,GeV}$. In all measured phase spaces, the Qgsjet-II approach shows large disagreements with the data as $p_{\text {T}}$ increases.

In Figs. 4c and 5c distributions of primary-charged-particle multiplicity, ${1}/{N_{\mathrm {ev}}} \cdot {{\mathrm {d} N_{\mathrm {ev}}}/{\mathrm {d} n_{\mathrm {ch}}}}$, are shown for minimum transverse momentum thresholds of $100 \mathrm {\, MeV}$ and $500 \mathrm {\, MeV}$, respectively. For the lower threshold, the distribution rises until values of $n_{\mathrm {ch}} \sim \!9$ before falling steeply. For the higher threshold the distribution peaks at $n_{\mathrm {ch}} \sim \!2$. None of the models are consistent with the data although the Epos LHC model provides a fair description. The two Pythia 8 calculations predict distribution peaks which are at higher $n_{\mathrm {ch}}$ than those observed and underestimate the event yield at low and high multiplicity. The Qgsjet-II tune overestimates the data at low and high $n_{\mathrm {ch}}$ values and underestimates the data for intermediate $n_{\mathrm {ch}}$ values.

The distribution of the average transverse momentum of primary charged particles, $\langle p_{\text {T}} \rangle$, versus the primary-charged-particle multiplicity, $n_{\mathrm {ch}}$, is given in Figs. 4d and 5d for transverse momentum thresholds of $100 \mathrm {\, MeV}$ and $500 \mathrm {\, MeV}$, respectively. The average $p_{\text {T}}$ rises with multiplicity although the rise becomes progressively less steep as the multiplicity increases. This is expected due to colour coherence effects in dense parton environments, which are modelled by a colour reconnection mechanism in Pythia 8 or by the hydrodynamical evolution model used in Epos. It is assumed that numerous MPI dominate the high-multiplicity events, and that colour coherence effects thereby lead to fewer additional charged particles produced with every additional MPI, which share a higher average $p_{\text {T}}$. The Epos LHC and Pythia 8 models provide a fair description of the data. The Qgsjet-II model fails to predict the mean transverse momentum over the entire multiplicity range, as it does not simulate colour coherence effects and therefore shows very little dependence on the multiplicity.

**Fig. 9:**
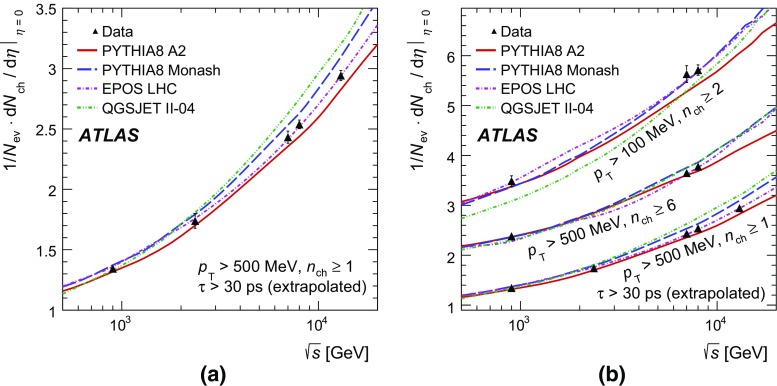
The average primary-charged-particle multiplicity per unit of pseudorapidity at $\eta = 0$ as a function of the centre-of-mass energy. Results are shown for the phase spaces **a** ($p_{\text {T}} > 500 \mathrm {\, MeV}$, $n_{\mathrm {ch}} \ge 1$) and **b** ($p_{\text {T}} > 500 \mathrm {\, MeV}$, $n_{\mathrm {ch}} \ge 1$), ($p_{\text {T}} > 500 \mathrm {\, MeV}$, $n_{\mathrm {ch}} \ge 6$), and ($p_{\text {T}} > 100 \mathrm {\, MeV}$, $n_{\mathrm {ch}} \ge 2$). The data are compared to various particle-level MC predictions. The results at $\sqrt{s} = 8$ and $13 \mathrm {\, TeV}$ are extrapolated to include strange baryons. The *vertical error bars* on the data represent the total uncertainty

**Table 1:** Central primary-charged-particle density ${1}/{N_{\mathrm {ev}}} \cdot {\mathrm {d} N_{\mathrm {ch}}}/{\mathrm {d} \eta }$ at $\eta = 0$ for five different phase spaces. The results are given for the fiducial definition $\tau > 300\,\mathrm{ps}$, as well as for the previously used fiducial definition $\tau > 30\,\mathrm{ps}$ using an extrapolation factor of $1.012 \pm 0.004$ (for $p_{\text {T}} > 100 \mathrm {\, MeV}$) or $1.025 \pm 0.008$ (for $p_{\text {T}} > 500 \mathrm {\, MeV}$), which accounts for the fraction of charged strange baryons predicted by Epos LHC simulation.

Phase space		${{1}}/{{N}_{{ev}}}\cdot {{d} {{N}}_{{ch}}}/{{d} {\eta }}$ at ${\eta } = 0$	
$n_{\mathrm {ch}} \ge$	$p_{\text {T}}$ >	$\tau > 300\,\mathrm{ps}$ (fiducial)	$\tau > 30\,\mathrm{ps}$ (extrapolated)
2	100 Mev	5.64 ± 0.10	5.71 ± 0.11
1	500 Mev	2.477 ± 0.031	2.54 ± 0.04
6	500 Mev	3.68 ± 0.04	3.78 ± 0.05
20	500 Mev	6.50 ± 0.05	6.66 ± 0.07
50	500 Mev	12.40 ± 0.15	12.71 ± 0.18

The evolution of the primary-charged-particle multiplicity per unit pseudorapidity at $\eta =0$ is shown in Fig. 9. It is computed by averaging over $|\eta |<0.2$ in the ${1}/{N_{\mathrm {ev}}} \cdot {\mathrm {d} N_{\mathrm {ch}}}/{\mathrm {d} \eta }$ distribution. In order to make consistent comparisons with previous measurements, these figures are corrected to the earlier $\tau > 30\,\mathrm{ps}$ definition of stable particles (to include the fraction of short-lived particles which have been excluded from this study), using a factor $1.012 \pm 0.004$ in the $p_{\text {T}} > 100 \mathrm {\, MeV}$ phase space and $1.025 \pm 0.008$ in the $p_{\text {T}} > 500 \mathrm {\, MeV}$ phase spaces, derived from predictions of the Epos LHC tune with uncertainties following comparisons of the predictions of different MC models. Results are shown for the phase spaces ($p_{\text {T}} > 500 \mathrm {\, MeV}$, $n_{\mathrm {ch}} \ge 1$), ($p_{\text {T}} > 500 \mathrm {\, MeV}$, $n_{\mathrm {ch}} \ge 6$), and ($p_{\text {T}} > 100 \mathrm {\, MeV}$, $n_{\mathrm {ch}} \ge 2$) along with available results from other ATLAS measurements at $\sqrt{s} = 0.9, 2.36, 7$ and $13 \mathrm {\, TeV}$ [14, 18, 23]. It can be seen that the total uncertainty in the measurement at $\sqrt{s} = 8 \mathrm {\, TeV}$ is about 30–40 % less than for the study with $7 \mathrm {\, TeV}$ data [18]. This was achieved due to our improved knowledge of the ID material distribution [39], which reduced the dominant source of systematic uncertainty by more than 50 % with respect to the previous $7 \mathrm {\, TeV}$ measurement. Predictions of various QCD-based models are also shown. The best description of the data is given by Epos LHC. The predictions of the Pythia 8 tunes provide a fair description of the shape of the multiplicity dependence with centre-of-mass energy. As in the case of the other presented distributions, calculations of Qgsjet-II give the worst description.

A full summary of central primary-charged-particle densities at $\eta =0$ in all measured phase spaces is given in Table 1, showing results obtained with the new as well as the previous fiducial definition.

## Conclusion

Measurements were made of distributions of primary charged particles produced in minimum-bias *pp* collisions at $\sqrt{s} = 8 \mathrm {\, TeV}$ with the ATLAS detector at the LHC. The results are based on a dataset corresponding to an integrated luminosity of $160~\upmu \mathrm{b}^{-1}$. Distributions of primary-charged-particle multiplicities as well as pseudorapidity and transverse momentum spectra are shown. With the fiducial definition of primary charged particles that was used in this study ($\tau > 300\,\mathrm{ps}$), the central primary-charged-particle multiplicity at $\eta = 0$ per event and unit of pseudorapidity was measured to be $5.64 \pm 0.10$ in events containing $n_{\mathrm {ch}} \ge 2$ primary charged particles with transverse momentum $p_{\text {T}} > 100 \mathrm {\, MeV}$, and $2.477 \pm 0.031$ in events with $n_{\mathrm {ch}} \ge 1$ and $p_{\text {T}} > 500 \mathrm {\, MeV}$. Using an extrapolation factor for short-lived charged particles with a lifetime between $30< \tau < 300\,\mathrm{ps}$, the central primary-charged-particle multiplicity was measured to be $5.71 \pm 0.11$ and $2.54 \pm 0.04$, respectively. The precision of these results is 30–40 % better than for the previous highest precision ATLAS measurements at 0.9 and $7 \mathrm {\, TeV}$. Compared with earlier studies, this paper also presents ATLAS measurements of final states at high multiplicities of $n_{\mathrm {ch}} \ge 20$ and $n_{\mathrm {ch}} \ge 50$. Predictions of various Monte Carlo models were compared with the data, and it was found that the best description is given by the Epos LHC tune, followed by the Pythia 8 A2 and Monash tunes. The measurements presented here are expected to provide valuable constraints for the tuning and further understanding of soft QCD physics models.

## Figures and Tables

**Fig. 10:**
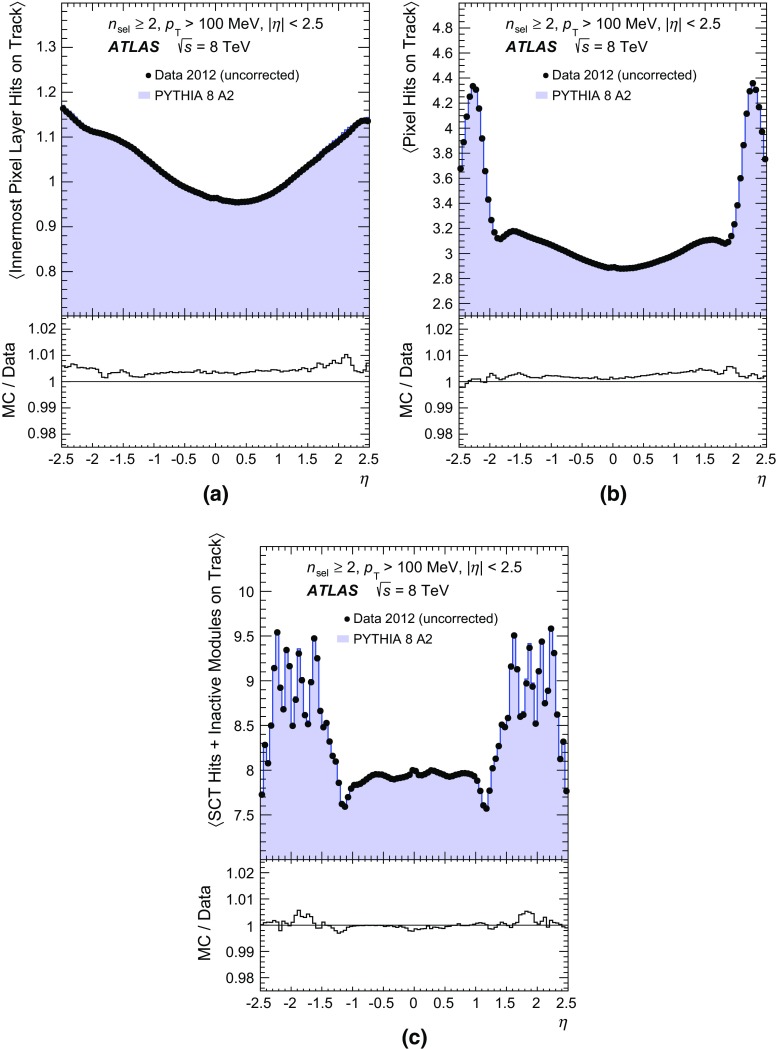
Data and MC simulation distributions of the average number of hits per reconstructed track as a function of pseudorapidity, $\eta$, in **a** the innermost layer of the Pixel detector, **b** the whole Pixel detector, and **c** the SCT detector (adding the average number of hits and inactive modules per track). The MC simulation distributions, made with Pythia 8 using the A2 tune, have been reweighted to match the reconstructed $p_{\text {T}}$ spectrum in data

**Fig. 11:**
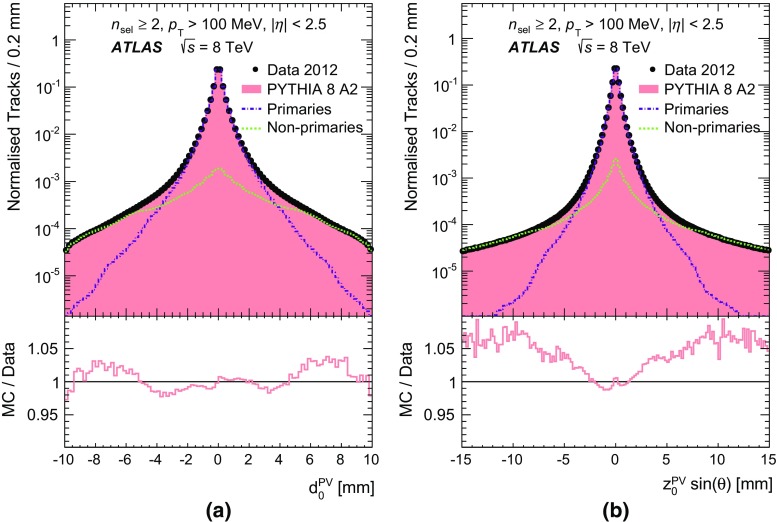
Normalised distributions of the **a** transverse and **b** longitudinal impact parameters of reconstructed tracks in data and MC simulation with respect to the reconstructed primary vertex, using events selected by the ($n_{\mathrm {sel}} \ge 2$, $p_{\text {T}} > 100 \mathrm {\, MeV}$) requirement. The MC simulation distributions, made with Pythia 8 using the A2 tune, have been reweighted to match the reconstructed $p_{\text {T}}$ spectrum as well as the fractions of reconstructed non-primary tracks in data

**Fig. 12:**
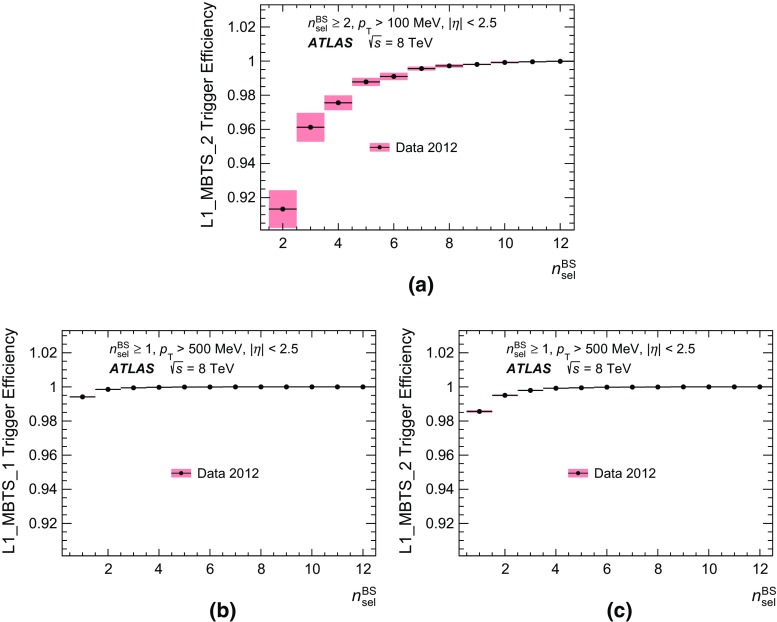
MBTS trigger efficiencies for $8 \mathrm {\, TeV}$ data in the two most inclusive measured phase spaces: **a** L1_MBTS_2 trigger efficiency as a function of the number of selected tracks $n_{\mathrm {sel}}^{\mathrm {BS}}$ in the phase space with $n_{\mathrm {sel}}^{\mathrm {BS}} \ge 2$ and $p_{\text {T}} > 100 \mathrm {\, MeV}$. **b** L1_MBTS_1 trigger efficiency as a function of the number of selected tracks $n_{\mathrm {sel}}^{\mathrm {BS}}$ in the phase space with $n_{\mathrm {sel}}^{\mathrm {BS}} \ge 1$ and $p_{\text {T}} > 500 \mathrm {\, MeV}$. **c** L1_MBTS_2 trigger efficiency as a function of the number of selected tracks $n_{\mathrm {sel}}^{\mathrm {BS}}$ in the phase space with $n_{\mathrm {sel}}^{\mathrm {BS}} \ge 1$ and $p_{\text {T}} > 500 \mathrm {\, MeV}$. L1_MBTS_1 and L1_MBTS_2 are the requirements that in at least one or two modules of the minimum-bias trigger scintillators a signal above threshold was registered, respectively. The *shaded areas* represent the sum of systematic and statistical errors

**Fig. 13:**
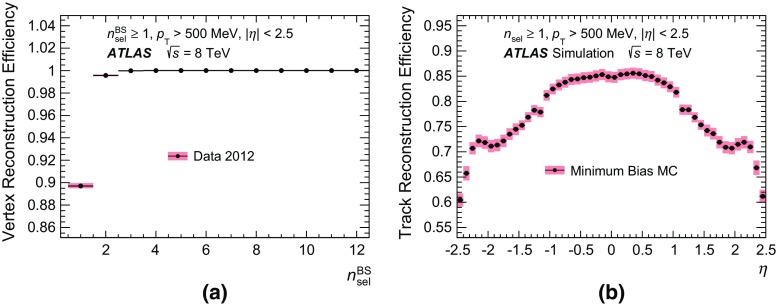
Selection efficiencies for $8 \mathrm {\, TeV}$ data in the $p_{\text {T}} > 500 \mathrm {\, MeV}$ phase space: **a** the vertex reconstruction efficiency as a function of the number of selected tracks, $n_{\mathrm {sel}}^{\mathrm {BS}}$. **b** The track reconstruction efficiency as a function of the pseudorapidity, $\eta$. The *shaded areas* represent the sum of systematic and statistical errors

## Average number of measurements per track

The distributions of the average number of hits per reconstructed track in data and MC simulation are shown in Fig. 10 for several detector components, using events selected by the ($n_{\mathrm {sel}} \ge 2$, $p_{\text {T}} > 100 \mathrm {\, MeV}$) requirement. The distributions are shown as a function of the pseudorapidity of the reconstructed tracks. The MC simulation distributions, made with Pythia 8 using the A2 tune, have been reweighted to match the reconstructed $p_{\text {T}}$ spectrum in data.

The level of agreement between data and MC simulation is found to be within ±1 % for the average number of measurements per track in the innermost layer of the Pixel detector (Fig. 10a), and remains within ±0.6 % for the average number of measurements per track in the whole Pixel detector (Fig. 10b) as well as the SCT (Fig. 10c). For the SCT, the sum of average measurements per track and inactive modules per track is shown. This is done in order to account for a mis-modelling of inactive modules in the MC simulation, which was shown to have negligible impact on other results presented in this paper.

## Distributions of impact parameters

The normalised distributions of transverse and longitudinal impact parameters of reconstructed tracks in data and MC simulation with respect to the reconstructed primary vertex, using events selected by the ($n_{\mathrm {sel}} \ge 2$, $p_{\text {T}} > 100 \mathrm {\, MeV}$) requirement, are shown in Fig. 11. The fractions of tracks originating from primary and secondary particles in the MC simulation (made with Pythia 8 using the A2 tune), which have been reweighted to match the reconstructed $p_{\text {T}}$ spectrum as well as the fractions of reconstructed non-primary tracks in data, are also shown.

The level of agreement between data and MC simulation is found to be within ±1.5 % in the signal region of selected tracks used in the analysis, where the impact parameters are within $\pm 1.5\,\mathrm{mm}$ of the reconstructed primary vertex. In the tail regions, the level of agreement remains within ±4 % for the transverse impact parameter and within ±9 % for the longitudinal impact parameter.

## Additional trigger efficiency plots

The efficiency of the trigger requirement in which the signal in at least one of the MBTS modules was above threshold is presented in Fig. 12b for the phase space with $n_{\mathrm {ch}} \ge 1$ and $p_{\text {T}} > 500 \mathrm {\, MeV}$. The efficiency of the trigger requirement in which the signal in at least two of the MBTS modules was above threshold is presented in Fig. 12a for the phase space with $n_{\mathrm {ch}} \ge 2$ and $p_{\text {T}} > 100 \mathrm {\, MeV}$, and in Fig. 12c for the phase space with $n_{\mathrm {ch}} \ge 1$ and $p_{\text {T}} > 500 \mathrm {\, MeV}$. All results are shown as a function of the number of selected tracks per event, $n_{\mathrm {sel}}^{\mathrm {BS}}$. In the phase space with the higher transverse momentum threshold, the trigger efficiency is higher and rises more quickly to 100 % for both trigger requirements.

## Additional vertex and track reconstruction efficiency plots

The vertex reconstruction efficiency for events selected in the phase space with the higher transverse momentum threshold, $p_{\text {T}} > 500 \mathrm {\, MeV}$, is presented in Fig. 13a as a function of the number of selected tracks per event, $n_{\mathrm {sel}}^{\mathrm {BS}}$. The systematic uncertainties are found to be small in comparison with those in the most inclusive phase space.

The track reconstruction efficiency for tracks from events selected by the $n_{\mathrm {sel}} \ge 1$ and $p_{\text {T}} > 500 \mathrm {\, MeV}$ requirement, which was determined from MC simulation using the Pythia 8 A2 tune, is presented in Fig. 13b as a function of the pseudorapidity.

## Footnotes

1 ATLAS uses a right-handed coordinate system with its origin at the nominal interaction point (IP) in the centre of the detector and the *z*-axis along the beam pipe. The *x*-axis points from the IP to the centre of the LHC ring, and the *y*-axis points upwards. Cylindrical coordinates $(r,\phi )$ are used in the transverse plane, $\phi$ being the azimuthal angle around the *z*-axis. The pseudorapidity is defined in terms of the polar angle $\theta$ as $\eta = -\ln \tan (\theta /2)$. Angular distance is measured in units of $\Delta R \equiv \sqrt{(\Delta \eta )^{2} + (\Delta \phi )^{2}}$.

2 The factor $2 \pi p_{\text {T}}$ in the $p_{\text {T}}$ spectrum comes from the Lorentz-invariant definition of the cross-section in terms of ${\mathrm {d}^3}p$. The results could thus be interpreted as the massless approximation to ${\mathrm {d}^3}p$.

3 A hit is a measurement point in a sensitive detector element which can be assigned to a reconstructed track.

4 In order to keep the readout rates from the MBTS trigger streams approximately constant during the run in which the data were collected, pre-scale factors that evolved during the run were applied for each trigger. Therefore, a combination of both L1 MBTS triggers was used to maximise the data yield and reduce statistical uncertainties.

5 The MC models used here were mostly tuned to data in the $p_{\text {T}} > 500 \mathrm {\, MeV}$ phase space (up to $\sqrt{s} = 7 \mathrm {\, TeV}$) and can therefore not necessarily be expected to describe the distributions at $p_{\text {T}} > 100 \mathrm {\, MeV}$ as well as at $p_{\text {T}} > 500 \mathrm {\, MeV}$.
